# On the Way to Become a Natural Killer Cell

**DOI:** 10.3389/fimmu.2019.01812

**Published:** 2019-08-02

**Authors:** Clara Di Vito, Joanna Mikulak, Domenico Mavilio

**Affiliations:** ^1^Unit of Clinical and Experimental Immunology, Humanitas Clinical and Research Center, Milan, Italy; ^2^Department of Medical Biotechnologies and Translational Medicine (BioMeTra), University of Milan, Milan, Italy

**Keywords:** natural killer cell, ontogenesis, hematopoietic stem cell, natural killer cell receptors, cytokines, self-tolerance, education

## Abstract

Natural Killer (NK) cells are innate lymphocytes playing pivotal roles in host defense and immune-surveillance. The homeostatic modulation of germ-line encoded/non-rearranged activating and inhibitory NK cell receptors (NKRs) determines the capability of these innate lymphocytes to either spare “self” cells or to kill viral-infected, tumor-transformed and heterologous cell targets. However, despite being discovered more than 40 years ago, several aspects of NK cell biology remain unknown or are still being debated. In particular, our knowledge of human NK cell ontogenesis and differentiation is still in its infancy as the majority of our experimental evidence on this topic mainly comes from findings obtained *in vitro* or with animal models *in vivo*. Although both the generation and the maintenance of human NK cells are sustained by hematopoietic stem cells (HSCs), the precise site(s) of NK cell development are still poorly defined. Indeed, HSCs and hematopoietic precursors are localized in different anatomical compartments that also change their ontogenic commitments before and after birth as well as in aging. Currently, the main site of NK cell generation and maturation in adulthood is considered the bone marrow, where their interactions with stromal cells, cytokines, growth factors, and other soluble molecules support and drive maturation. Different sequential stages of NK cell development have been identified on the basis of the differential expression of specific markers and NKRs as well as on the acquisition of specific effector-functions. All these phenotypic and functional features are key in inducing and regulating homing, activation and tissue-residency of NK cells in different human anatomic sites, where different homeostatic mechanisms ensure a perfect balance between immune tolerance and immune-surveillance. The present review summarizes our current knowledge on human NK cell ontogenesis and on the related pathways orchestrating a proper maturation, functions, and distributions.

## Introduction

Natural Killer (NK) cells were first described as large granular lymphocytes with a natural ability to kill tumor cells without a previous activation (1). Currently, it is well-known that NK cells mediate immune-surveillance not only via cytotoxic effector-functions, but also by serving as regulatory lymphocytes able to secrete cytokines and to interact with both innate and adaptive immune cells, such as monocyte/macrophages, dendritic cells (DCs), and T lymphocytes (2–5). These activities are governed by a balance between activating and inhibitory NK cell receptors (aNKRs and iNKRs) expressed on cell surface (6–9). Under homeostatic conditions, NK cells remain in a resting state due to the engagement of iNKRs [i.e., inhibitory Killer Immunoglobulin-like receptors (iKIRs), the C-type lectin receptor NKG2A, Ig-like transcripts (ILTs), and the leukocyte Ig-like receptors (LIRs)], that recognize a broad spectrum of classical and non-classical Human Leukocyte Antigen (HLA)-I molecules constitutively expressed of autologous “self” cells (10, 11). Viral infected, tumor-transformed or allogeneic “non-self” cells down-regulate, lack or express different HLA-I alleles, thus boosting the NK cell-mediated killing of these dangerous targets via the engagement of aNKRs, that includes Natural Cytotoxicity Receptors (NCRs) (NKp30, NKp46, and NKp44), C-type lectin receptors (NKG2C, NKG2D), DNAM-1 and activating KIRs (aKIRs) (“missing self hypothesis”) (6, 12–14).

NK cells comprise two main subsets defined on the basis of CD56 and CD16 surface expression: the cytotoxic CD56^dim^/CD16^pos^ (CD56^dim^) population accounting for up to 90% of circulating NK cells and the regulatory CD56^bright^/CD16^neg^ (CD56^br^) NK cell subset producing high amount of pro-inflammatory cytokines, such as interferon (IFN)-γ. These two NK cell populations also differ for the expression of several NKRs that determine their ability to respond to different stimuli (15). Both genetic and environmental (i.e., infections and microbes) factors also contribute to generate NK cell diversity in terms of NKR repertoire and functions (16). Indeed, extensive flow-cytometry and mass-spectrometry data clearly showed that a large diversity in the phenotype of NK cell subsets can coexist especially at tissue levels (17–20). This heterogeneity is also associated with the different microenvironments in which NK cells develop and reside (21). However, although these cells are widely distributed in several tissues and organs of human body, most of the current knowledge on these innate lymphocytes is limited to peripheral blood (PB-) NK cells (22). In this context, how and to what extent NK cells are exchanged between blood and human tissues and which anatomic compartments host tissue-resident NK cells represent important matters of scientific debate.

In this review, we discuss our current knowledge of the several steps of human NK cell ontogenesis with a special focus on those related mechanisms regulating their development, tissue homing and residency.

## Tissue Sites of Natural Killer Cell Development

The production and the maintenance of NK cells in the blood are sustained by CD34^pos^ hematopoietic stem cells (HSCs). However, the exact sites of NK cell development are poorly defined, as hematopoietic cell precursors have been found in different anatomic compartments of the human body both in intra-uterine and adult lifespan (23). In the embryo and fetus the hematopoiesis takes place in the yolk sac, aorta-gonad-mesonephros region, and liver, while in adults bone marrow (BM), thymus, spleen, omentum, and liver are considered the main sites of blood cell development (24).

BM has been considered for long time the major site of NK cell generation and differentiation after birth. Indeed, this immunological niche is highly enriched of CD34^pos^ HSCs and hematopoietic progenitors, including NK cell ones (25). Herein, NK cell development is supported through interactions with stromal cells, cytokines, growth factors, and other soluble molecules. However, whether NK cell ontogenesis occurs exclusively or primarily in the BM niche is still being debated.

In this regard, tissue-specific NK cell development had been reported and even the so-called NK cell “education” ensuring self-tolerance can occur in certain tissues (25). Indeed, several lines of evidence demonstrated that, while the early phases of NK cell development occur in the BM, later stages of NK cell differentiations can take place in secondary lymphoid tissues (SLTs), PB, liver, mucosa-associated lymphoid tissues (MALTs), and uterus (22, 26–29). In particular, tonsils, spleen, and lymph nodes (LNs) are considered those SLTs hosting the main extra-medullary sites of NK cell development and maturation. The para-follicular T cell regions of LNs are one of the main anatomical districts enriched with NK cells. Here, more than the 90% of tissue-resident NK cells have a CD56^br^ phenotype and they are able to differentiate in mature CD56^dim^ NK cells following stimulation with interleukin (IL)-2, as circulating CD56^br^ NK cells (15, 30). These findings suggest that LNs might be one of the major peripheral tissue sites of NK cell development. This working hypothesis is further corroborated by other evidences showing that human LNs contain CD34^dim^/CD45RA^br^ hematopoietic precursors that likely origin from HSCs in the BM and then traffic in this SLT via the PB. Once in LNs, they can differentiate in CD56^br^ NK cells upon activation mediated by LN-resident T cells (26).

The existence of CD34^pos^ lymphoid precursors endowed with the ability of differentiating in NK cells *in vitro* have been also reported in human thymus (31). However, patients either affected by the Di George syndrome (32) or undergone thymectomy (33, 34) and splenectomy (35) have normal frequencies of circulating NK cells, that are also phenotypically and functionally similar to those of healthy donors (30). For that reason, thymus as well as spleen are not considered major sites of NK cell ontogenesis. Although it is possible that these unaltered frequency, phenotype and functions could be due to the redundancy of NK cell developmental pathways.

Fetal liver certainly represents one of the major tissue of NK cell development and this solid organ also retain a residual ability to generate NK cells even after birth (36). Indeed, human liver is highly enriched in tissue-resident NK cells that are phenotypically and functionally distinct from their circulating counterparts (29, 37–39). Moreover, it has been reported that human liver perfusates and biopsies contain all NK cell developmental stages from multipotent CD34^pos^ hematopoietic progenitors to terminally differentiated cells. In addition, liver-resident NK cell precursors retain the ability to generate *in vitro* fully mature and functional NK cells (29). Taken together, these data further support the hypothesis that adult liver represents an important tissue site for NK cell development *in vivo* even in the adult life.

Another peripheral organ highly enriched of tissue-resident NK cells is the uterus. Here, the so-called uterine NK (uNK) cells hold a unique phenotypic/functional profile and they are present at high frequencies in the decidua to ensure mother tolerance vs. the implanted fetus. uNK cells play also a primary role in angiogenesis, tissue remodeling, and immune modulation mainly during the first trimester of pregnancy (40–42). In this regard, a population of CD34^pos^ cells able to differentiate in NK cells either following *in vitro* stimulation with several cytokines or upon co-culture with decidual stromal cells had been described in human decidua (43, 44).

Although ~10–20% of total lymphocytes in human lungs are NK cells, they share a very similar phenotype with circulating CD56^dim^ NK cell subset and express very low levels of tissue-residency markers. This observation thus suggests that lung NK cells, different from liver and uterus, likely migrate in this tissue from the PB (21).

## Natural Killer Cell Precursors and Ontogenesis

Our current knowledge on immune cell hematopoiesis postulates that the earliest step of HSCs to undergo the NK cell differentiation relies on their commitment toward the lymphoid/myeloid lineage rather than the erythroid/megakaryocyte one. Then, CD34^pos^/CD133^pos^/CD244^pos^ cells acquire the expression of CD45RA to become Common Lymphoid Progenitors (CLPs), which have the potential to generate B, T and innate lymphoid cells (ILCs) (45). This process requires cell-to-cell interactions with stromal cells in the context of a peculiar microenvironment characterized by the presence of the stem cell factor (SCF), the ligand for the fms-like tyrosine kinase 3 (FLT3L), and IL-7 (46).

**Figure 1:**
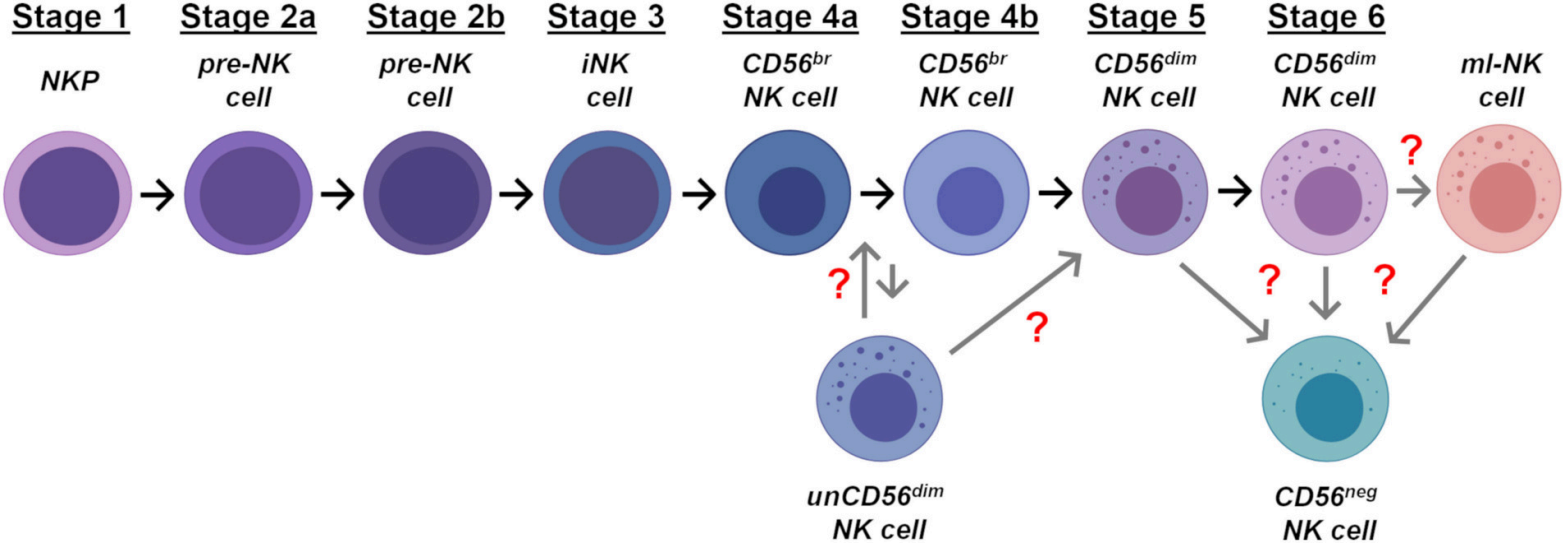
Stages of NK cell ontogenesis. Schematic representation of the different stages of NK cell differentiation in human bone marrow and secondary lymphoid tissues. Gray arrows and red question marks indicate the possible location in NK cell development of unCD56^dim^, CD56^neg^, and ml-NK cells.

CLPs can then further differentiate in NK cell progenitors (NKPs) that are classified in three sequential stages of maturation named NK cell progenitors (stage 1), pre-NK cells (stage 2), and immature NK (iNK) cells (stage 3) (Figure 1) (47, 48). The commitment of CLPs toward NKPs had been first postulated for analogy with B and T cells progenitors and it is characterized by the down-regulation of CD34 and by the acquisition of CD122, the common IL-2 receptor subunit β shared by IL-2 and IL-15 signaling pathways. The induced expression of CD122 marks the irreversible fate of CLPs toward the NK cell differentiation (22, 49, 50). Indeed, both NKPs and pre-NK cells still express CD34 and retain the ability to differentiate in T cells, DCs and other ILCs. On the opposite, CD34^neg^/CD122^pos^ iNK cells loose this development potential, thus representing the real NKPs (Table 1) (47, 51).

**Table 1:** Principal surface markers differentially expressed on NK cell developmental intermediates.

**Surface marker**	**Stage 1**	**Stage 2a**	**Stage 2b**	**Stage 3**	**Stage 4a**	**Stage 4b**	**Un CD56^**dim**^**	**Stage 5**	**Stage 6**	**ml-NK**	**CD56^**neg**^**
*CD34*	+	+	+	–	–	–	–	–	–	–	–
*CD10*	+	+/–	+/–	–	–	–	n.d.	–	–	–	–
*HLA-DR*	+	+	+	–	–	–	n.d.	–	–	+	+
*CD117*	–	+	+	+	+/low	low/–	–	–	–	–	–
*CD127*	+	+	+	+	–	–	–	–	–	–	+
*CD45RA*	+	+	+	+	+/–	+/–	n.d.	–	–	–	–
*IL-1 βR*	–	–	+	+	+/low	low/–	n.d.	low/–	low/–	low/–	–
*CD122*	–	–	+	+	+	+	n.d.	+	+	+	+
*CD161*	–	–/low	–/low	–/+	+	+	n.d.	+	+	low/–	+
*CD56*	–	–	–/low	–/low	++	++	+	+	+	+	–
*CD94*	–	–	–	–	+	+	+	+/–	+/–	+	+
*NKG2A*	–	–	–	–	+	+	+	low/–	low/–	low/–	low/–
*NKG2D*	–	–	–	–/low	+	+	+	+	+	+	+
*NKp30*	–	–	–	–/low	+	+	+	+	+	low/–	low
*NKp46*	–	–	–	–/low	++	++	–/low	+	+	low	low
*NKp80*	–	–	–	–	–	+	n.d.	+	+	+	+
*NKG2C*	–	–	–	–	low/–	low/–	low/–	+	+	++	+/low
*CD16*	–	–	–	–	–	–	–	+	+	+	+
*KIRs*	–	–	–	–	–	–	low	+	+	+	+
*CD57*	–	–	–	–	–	–	–	–	+	+	+

*n.d., not determined*.

More recently, two distinct and additional stages of pre-NK cells have been described on the basis of their negative (stage 2a) or positive (stage 2b) expression of both IL-1β and IL-2β receptors (Figure 1 and Table 1). Stage 2a is mainly enriched in certain tissues (i.e., SLTs and PB) and retains the ability to give rise to T cells and DCs, while stage 2b represents the so-called common ILC progenitors, since its commitment is restricted to the generation of ILCs, including NK cells (48, 52). The transition from stage 2b to stage 3 is then marked by the acquisition of aNKR expression (i.e., NKG2D, NKp30, and NKp46) (Table 1).

All the developmental stages of NKPs have been mainly characterized in the context of the BM niche. However, it is still an important matter of debate whether distinct organ-specific NKPs also exist and could undergo a “peripheral ontogeny” able to generate tissue-resident NK cells (25). In this regard, a subset of putative NKP has been recently identified in BM, PB and SLTs, where it can give rise to all members of ILC lineage. Differently from the above-mentioned stage 2b pre-NK cells, these latter NKPs are characterized by a CD34^pos^/CD45RA^pos^/CD38^pos^/CD10^pos^/CD7^pos^/CD123^neg^ /CD127^neg^ phenotype sharing several surface markers with both stage 1 and stage 2 NKPs (53). Finally, an additional CD56^pos^ subset of CD34^neg^/CD117^pos^ precursors able to generate NK cells and ILC3s, but not ILC2s, has been described in tonsils (54).

In our currently accepted linear model of maturation (Figure 1), the sequential expression of specific markers on the surface of iNK cells (stage 3) parallels the acquisition of NK cell self-tolerance and effector-functions. In particular, the shift from NKPs to mature NK cells is associated with the sequential acquired expression of CD56, CD94, and of the Killer C-type lectin receptor CD161 (55). While the functional roles of the expression of both CD161 and CD56 have not yet been fully clarified, the acquisition of CD94 surface expression is essential to allow the formation of the heterodimeric C-type Lectin receptors. Hence, the CD34^neg^/CD117^pos/neg^/CD94^pos^/HLADR^neg^/ CD10^neg^/CD122^pos^/CD94^pos^/NKp44^low^/NKG2D^pos^/CD161^pos^ phenotype defines mature NK cells that can be then further distinguished into in the 2 final developmental stages according to the expression of CD56 and CD16 (Table 1) (25, 56, 57).

CD56^br^ NK cell represents the immune-regulatory and cytokine producer stage 4, characterized by a CD34^neg^/CD117^low^/CD94^pos^/CD16^neg^ phenotype (Table 1). More recently, 2 distinct stages 4 of NK cells have been described in SLTs: 4a and 4b stages that differ for the induced expression of NKp80 on the latter subset (58, 59). The stage 4a NKp80^neg^/CD56^br^ NK cell subset is characterized by constitutive high expression of NKG2D, NKp30, and NKp46, CD94/NKG2A, CD161, and is not endowed with potent effector-functions (Table 1). On the opposite, its stage 4b counterpart can produce IFN-γ and mediate perforin-dependent cytotoxicity *in vitro* (48). Both 4a and 4b NK cell stages are then considered precursors of the terminally-differentiated and cytotoxic CD56^dim^ NK cells (stage 5) (25) (Figure 1). Indeed, the CD56^br^ NK cell subset does not express KIRs and CD57 and it is more immature as also confirmed by the longer length of its telomeres (60). Additional experimental evidence demonstrated that the transition from CD56^br^ to CD56^dim^ NK cells is progressive as the latter terminally-differentiated subset gradually acquires the expression of CD16, KIRs, and cytotoxic granules by generating a transitory population of CD56^bright^/CD16^pos^ NK cells (61). During this transition, stage 4 CD56^br^ NK cells lose the expression of CD117, CD127, and CD94/NKG2A receptor, while acquiring CD94/NKG2C and down-regulating CD56 (60, 62) (Table 1). Finally, it has been also recently proposed that stage 5 CD56^dim^/KIR^pos^ NK cells can be further distinguished from stage 6 based on the expression of CD57, a surface marker of replicative senescence (Figure 1 and Table 1). Although this is still a matter of scientific discussion, a recent study confirmed at transcriptome, epigenome, and proteomic levels that this linear developmental trajectory starts from CD56^br^ NK cells and ends with the final acquisition of CD57 (63).

## Additional Stages of NK Cell Maturation

### Memory-like NK Cells

While stage 6 CD56^dim^ NK cells show a poor responsiveness to cytokine stimulation, they retain high degree of cytotoxicity and can expand in response to several viral infections (64, 65). In this regard, it has been shown that some viruses can change the NKR repertoire and can also induce the clonal expansion of peculiar NK cell subsets endowed with adaptive features. These latter populations display higher effector-functions when re-encountering the same virus and they are defined “memory-like” NK (ml-NK) cells (Figure 1) (16, 19, 66, 67). ml-NK cells are characterized by a peculiar KIRs^pos^/CD57^pos^/NKG2C^pos^ phenotype and lack the expression of CD161, NKp30, and CD7 (68–70). Among the main viruses inducing the expansion of these NK cells endowed with adaptive traits there is the Human Cytomegalovirus (HCMV) that drives a profound epigenetic reprogramming in ml-NK cells. This HCMV-driven mechanism increases the IFN-γ production when ml-NK cells are re-exposed to the same viral pathogen (71–75).

ml-NK cells have been described not only in PB but also in tissues and associated with different antigens. Indeed, a subset of a hepatic CXCR6^pos^ NK cells with adaptive properties against haptens or viral antigens had been also reported. This latter subset of ml-NK cells is liver-resident and express a CD49a^pos^/DX5^neg^ phenotype (76, 77).

### CD56^neg^ NK Cells

Besides the induced expansion of ml-NK cells, viral infections can also drive the emergence of another dysfunctional CD56^neg^/CD16^pos^ (CD56^neg^) NK cell subset. These cells had been firstly described during the course of HIV-1 infections and then in other viral diseases, in autoimmune-disorders and in elderly. CD56^neg^ NK cells are present at very low frequency in the PB of healthy subjects, but they represent the majority of total NK cells in AIDS patients showing high levels of HIV-1 viremia (74, 78–81). Despite being identified and characterized more than 2 decades ago, the ontogenesis of this subset is still unknown. The repertoire of NKRs expressed on CD56^neg^ NK cells shared several similarities with that of stage 3 iNK cells. However, the high constitutive expression of CD94/NKG2A, NKG2D, and CD16 together with the retention of a certain degree of cytotoxicity represent phenotypic and functional differences that do not allow a completely overlap between CD56^neg^ NK cells and stage 3 iNK cells (Table 1) (79, 82). Indeed, the high surface levels of KIRs, CD57 and CD107a degranulation marker on CD56^neg^ NK cells suggest that they rather represent exhausted lymphocytes that already engaged target cells (Figure 1) (83).

### Unconventional CD56^dim^ NK Cells

The latest NK cell subset to be identified and characterized is represented by the so called unconventional NK cells holding a CD56^dim^/CD16^neg^ phenotype (unCD56^dim^) (84). This neglected population is extremely rare under homeostatic conditions, although it displays a significantly higher cytotoxicity compared to that of CD56^br^ and CD56^dim^ NK cell subsets. However, a very few studies characterized the homeostasis, the phenotype and the functional relevance of unCD56^dim^ NK cells subset although there is not yet a consensus on its name and classification (85–89). Unexpectedly, other and we recently reported that, in the context of the lymphopenic environment of patients affected by hematologic malignancies and undergone haploidentical stem cell transplantation (haplo-HSCT), unCD56^dim^ NK cells are by far the largest subset of NK cells immune-reconstituting in the first 2–4 weeks after the transplant (88, 90). Indeed, in this short window after haplo-HSCT the very low frequency of the conventional cytotoxic CD56^dim^ NK cells are compensated by the high expansion of unCD56^dim^ NK cells that lack the expression of CD34, CD117, and CD127 (Table 1). These data demonstrate that unCD56^dim^ NK cells cannot be classified as NKPs, but are rather differentiated cells expressing several NKRs as well as lytic granzyme and perforin. Moreover, the transcriptional profile of unCD56^dim^ NK cells revealed that they are placed within an intermediate stage of differentiation between CD56^br^ and CD56^dim^ NK cells as also functionally assessed with time-course *in vitro* experiments of NK cell differentiation (88). Furthermore, those unCD56^dim^ NK cells highly expanded early after haplo-HSCT also have a transient high expression of CD94/NKG2A, an iNKR also involved in NK cell differentiation. This phenomenon makes this subset anergic only in this particular human setting *in vivo*. Hence, the use of an immunotherapeutic strategy to block this inhibitory checkpoint, unleashing NK cells thus improving the clinical outcome of haplo-HSCT early after the infusion of HSCs is currently under clinical investigation (84). Taken together, these data highlight the key role played by unCD56^dim^ NK cells in the mechanisms of immune-reconstitution and also show that this unconventional NK cell subset could represent and additional or alternative stage of NK cell differentiation (Figure 1).

## Signals and Mechanisms Regulating the Differentation of NK Cells

### Cytokines

NK cell differentiation is finely tuned by different cytokine signals (48, 91). As previously mentioned, HSC survival and proliferation are preserved by FLT3L and SCF. Indeed, mice lacking their receptors FLT3 and c-Kit (CD117) show a consistent reduction in the frequency of CLPs (46, 92–94). In addition, the engagements of FLT3/FLT3L and c-Kit/SCF axes induce the expression of CD122 and/or IL-15Rα (CD215), thus increasing the sensitivity of NKPs to IL-15 (51, 95). Although both IL-15 and IL-2 stimulation promote the maturation of CD56^br^ toward CD56^dim^ NK cells *in vitro* (96), only IL-15 is involved in NK cell differentiation both in humans and mice.

This is confirmed by the experimental evidence showing that mature NK cells are nearly absent in mice lacking any of the 3 different subunits that compose the IL-15 heterotrimeric receptor (IL-15R) complex: CD215, CD122, and CD132 (γ_c_ chain) (97–101). Accordingly, patients showing an X-linked gene mutation in the γ_c_ gene (*il2rg*) are affected by a severe combined immune-deficiency characterized by a high susceptibility to infections due to developmental defects of lymphocytes (including NK cells) (100). Despite the γ_c_ chain of IL-15R is shared by other relevant cytokines (i.e., IL-2, IL-4, IL-7, IL-9, and IL-21) for their downstream signaling (102), dysfunctions of this subunit affects only the IL-15 pathway. Indeed, knockout mice lacking IL-2, IL-2Rα, IL-7, IL-7Rα, and IL-21R have normal frequencies of mature circulating NK cells (103–106).

The production of IL-15 at NK cell developmental site is mainly exerted by BM stromal and myeloid cells (107). The binding of soluble IL-15 to CD215 on the surface of surrounding cells mediates the trans-presentation of this complex to NK cells expressing CD122 and CD132 heterodimer (108–110). This engagement of IL-15R on NK cells induces the activation of JAK1/3 downstream cascade that, in turn, activates STAT3/5 and the mitogen-activated protein kinase (MAPK). These signaling pathways mediate NK cell survival via both the up-regulation of anti-apoptotic B cell lymphoma 2 (BCL-2) family members and the down-regulation of pro-apoptotic proteins (111–114). Indeed, both *Stat5*-deficient and NK cell-specific *Stat5*-deficient mice show a marked reduction of circulating mature NK cells (115, 116). In humans, a similar severe reduction in NK cells is observed in patients with a mutation of *STAT5b*, one of the two highly conserved *Stat5* human genes (117, 118).

NK cell responsiveness to IL-15 during NK cell development is also influenced by the expression of phosphoinositide-dependent kinase-1 (PDK1) that connects IL-15 signaling to the activation of both E4BP4 (also known as Nfil3) and Inhibitor of DNA-binding 2 (ID2) transcription factors (119–121).

An additional cytokine playing a critical role in the development of NK cells is IL-21. Indeed, IL-21 stimulation *in vitro*, together with FLT3 and IL-15, promotes the differentiation and the expansion of cytotoxic CD56^dim^ NK cells from BM progenitors (104, 122). In addition, IL-21 induces rapid maturation of human NK cells and the acquisition of a KIR^pos^ mature phenotype from CD34^pos^ cell precursors (123). On the other side, IL-7 is key in promoting the survival and early differentiation of NKPs (106). As a matter of fact, although mice lacking IL-7 or its receptor (CD127) keep a relatively normal NK cell development (105), the correct engagement of CD127 expression is key in the early stages of NK cell differentiation and in the retention of NKPs in SLTs (52). Moreover, those NK cells enriched in thymus are characterized by high constitutive expression of CD127 and require IL-7 for their homeostasis (100).

IL-4 has been recently described of being able to induce the development of tissue-resident NK lymphocytes in mice by converting CD49a^pos^/Eomes^neg^ NK cells into their functional CD49a^pos^/Eomes^pos^ counterparts (124). Since CD49a^pos^/Eomes^neg^ NK cell subset is considered a liver-resident NK cell subset in mice, these findings could be relevant for a better understanding of the specific tissue-resident generation of NK cells (125). However, other than expressing high levels of Eomesodermin (Eomes) transcripts, human liver-resident NK cells appear to be much more phenotypically heterogeneous compared to their murine counterparts (126).

IL-12 can also promote differentiation of NK cells and can enhance their cytotoxicity *in vitro* (127). Interestingly, an alternative pathway of NK cell development that bypasses the above-mentioned γ_c_-signaling relies on the engagement of IL-12 in response to viral infections. Indeed, the stimulation of NKPs with this pro-inflammatory cytokine in the BM generates an unconventional but yet functional NK cell subset. However, this pathway is still not exploited in humans and might be highly relevant in patients with SCID (128). IL-12, together with IL-18, has been also described for its ability to induce the differentiation of ml-NK cells. In this regard, IL-12 and IL-18 have been studied as co-stimulatory factors for the generation of CMV-specific murine Ly49H^pos^ ml-NK cells (129). In particular, the IL-12/STAT4 signaling pathway is required for the formation and the expansion of these NK cells with adaptive traits (130). Similarly, the expansion of NKG2C^pos^ ml-NK cells in humans upon HCMV infection has been shown to be IL-12- plus IL-18-dependent (131, 132). In this regard, *in vitro* activation of both murine and human NK cells with IL-12, IL-18, and IL-15 supports the generation of cytokine-induced ml-NK cells (133, 134). This mechanism has been recently employed in a clinical trial to boost the expansion of adaptive NK cells showing enhanced anti-tumor responses against myeloid leukemia (135).

### Transcription Factors

The commitment and differentiation of hematopoietic stem cells/precursors toward NK cell lineage require the expression of specific transcription factors (TFs). In this context, the current knowledge on NK cell development derives from experimental findings mainly generated either *in vitro* or in animal models and very little is known in human setting *in vivo*. However, it is widely accepted that several TFs are required by CLPs for their transition to both NKPs and iNK cells. These mechanisms are not specific for NK cell development as the same TFs are used to commit CLPs toward different cell lineages (136, 137). Ets-1 and PU.1, members of the Ets TF family, are involved in the transition of CLPs to NKPs and they are broadly expressed in multiple hematopoietic-derived lineages (138–141). Interestingly, Ets-1-deficient mice have a severe decrease of circulating NK cells, while knocking out PU.1 in murine models does not affect the frequency of NK cells in PB. These latter animals are also characterized by an up-regulation of Ets-1, thus suggesting the existence of compensatory mechanisms to ensure a correct ontogenesis and maturation of NK cells (142, 143).

As previously mentioned, the transition to NKPs also requires the expression of CD122 that induces STAT5 phosphorylation, dimerization and nuclear translocation (115–117). Although the specific gene targets of STAT5 in NK cells have not yet been clarified, more than 15,000 STAT5 DNA binding sites have been identified in T cells, including genes required for lymphocyte proliferation and survival (144). In addition, the expression of CD122 in NK cells is regulated by the Runx family of TFs that represent key regulators of lymphocyte lineage-specific gene expression (145, 146). In particular, Runx3 has been reported to play an important role both in NK and CD8^pos^ T cell development, thus indicating its specific involvement in transcriptional programs of cytolytic lymphocytes (146). Similarly, Thymocyte selection-associated high mobility group box protein (Tox) and the interferon-regulatory factor (IRF) families regulate the transition toward NKPs as well as toward B and T cells, ILCs and myeloid lineages (147–149). On the contrary, several other TFs regulating the early steps of NK cell differentiation are much more restricted to the development of innate lymphocytes. Indeed, E4BP4-deficient mice lack only NK cells and ILCs, as the expression of this TF is required to tune the expression of Eomes and ID2 in early progenitor cells (150–154). Other reports also claimed the existence of alternative and E4BP4-independent development pathways for immature and tissue-resident NK cells (125, 155, 156).

Another TF required for the differentiation of ILCs is ID2, whose expression is controlled by Ets-1 (138). ID2-deficient mice show a block of NK cell development between NKPs and mature NK cells with the subsequent lacking of circulating NK cells (120, 121). Recently, it has been also reported that ID2 regulates NK cell responsiveness to IL-15 through the modulation of DNA-binding helix-loop-helix E proteins (E2A) (157).

In later stages of NK cell maturation, T-box protein 21 (T-bet) and Eomes play a major role in promoting the expression of cytolytic and IFN-γ production machineries (158, 159). Indeed, mice deficient for both these latter TFs have a systemic lack of circulating mature NK cells (160, 161). However, whether or not Eomes and T-bet act in the same pathway is not yet clear as these two members of the T-box family are believed to function in a sequential manner during NK cell maturation. T-bet is required for the production of iNK and it is detectable just prior this development stage (154). Indeed, T-bet deficiency results in an accumulation of iNK cells in BM (161, 162). On the contrary, Eomes tunes the differentiation of mature NK cells from iNK cells and it is also critical to discriminate between NK and ILC1 subsets (158, 159). In addition, T-bet and Eomes have been reported to regulate NK cell development at different anatomical site as T-bet is primarily required for the production of NK cell at extramedullary sites (125, 163, 164).

## Surface Molecules Regulating NK Cell Trafficking and Maturation

Very little is known about the mechanisms orchestrating the trafficking of human NKPs and mature NK cells from PB to tissues/organs and *vice versa* (165). Several lines of evidence indicate that this trafficking is governed by several adhesion molecules, such as integrins, selectins, and chemokine/cytokine receptors. Among them, CXCR4, the alpha-chemokine receptor specific for the stromal derived factor-1, has a role in maintaining HSCs in the BM niche (166–168). Indeed, it has been shown that the treatment with a CXCR4 antagonist promotes the progenitor mobilization from the BM (169, 170). CXCR4 appears to also play a key role in the first steps of NK cell ontogenesis since it is highly expressed by NKPs and iNK cells, while its surface levels gradually decrease during NK cell maturation (171).

Differently from CXCR4, the down-modulation of CX3C chemokine receptor 1 (CX3CR1) in response to stimulation with transforming growth factor (TGF)-β prevents the NK cell egress from the BM (172, 173). Furthermore, CX3CR1, together with CC chemokine receptors (CCRs)-7 and−5, tunes NK cell maturation as the acquisition of a CD56^dim^ phenotype is associated to its induced expression (174, 175). Similar to CX3CR1, the sphingosine-1-phosphate receptor 5 (S1P5) is involved in the NK cell release in the bloodstream and in NK cell differentiation. Indeed, terminally differentiated stage 6 NK cells up-regulate S1P5 and migrate in response to sphingosine-1-phosphate (S1P) (176). The active role of this bioactive sphingolipid in determining the NK cell trafficking is also suggested by the observation that S1P creates a gradient with highest concentrations in the blood and lymph, while its levels are maintained low in tissue parenchyma (177).

Several other adhesion molecules and chemokine receptors regulate the preferential localization of CD56^br^ and CD56^dim^ NK cells in SLTs, PB and inflamed tissues (178). Indeed, while S1P5 seems to be involved in retaining CD56^dim^ NK cells in the bloodstream, CD62L, CCR7, and CXCR3 are involved in the selective homing of CD56^br^ NK cell to LNs. Indeed, these latter surface molecules are either absent or expressed at very low levels on CD56^dim^ NK cells (30, 62, 179, 180). Furthermore, CD69 is now considered not only as a marker of cell activation, but also as a tissue-residency one. Indeed, CD56^br^ NK cells in tissues (i.e., liver, uterus, LNs) express high levels of CD69, while their counterparts in PB are CD69^neg^ (38, 181). Moreover, highly cytotoxic CD56^dim^ cells infiltrating metastatic LNs express CD69 and CCR7 and can upregulate CXCR1 (182–184). CD103 and CD49a are other tissue-residency markers that are up-regulated by NK cells in response to TGF-β (185, 186). CD103 heterodimerized with β7 and binds to E-cadherin on epithelial cells, thus retaining NK cells in tissues (187). Moreover, the heterodimer β1-CD49a is involved in the tissue retention of NK cells via the binding to collagen (188). Those CD56^dim^ NK cells preferentially migrating toward inflamed tissues, express a different patterns of cytokine/chemokine receptors that include CXCR1, CXCR2, and ChemR23 (59, 175, 189).

## NK Cell Education and Acquisition of Tolerance to Self

Although NK cell ontogenesis and education are two separated processes, there are quite a few interconnections between these two key mechanisms of NK cell homeostasis. The acquired expression of iNKRs together with their binding to self-HLA molecules in BM during NK cell development represent the mechanism generating functional NK cells that are also tolerant against autologous targets (190). Indeed, the direct cell-to-cell interactions with “self”-MHC-I educate NK cells to sense the down-regulation or lack of matched HLA alleles on target cells in order to mount an efficient effector-responses only against threatening viral-infected or tumor-transformed or allogeneic targets (190–192). Hence, the so-called process of “NK cell education” relies on the avidity of binding between NKRs and self-HLA molecules and on the level of response of an NK cell to activating signals (i.e., stress ligands, inflammatory cytokines, and Fc receptor engagement) (193, 194).

In addition to the recognition of self-HLA/MHC antigens in *trans* on neighboring cells, the expression of MHC-I molecules on the NK cell itself has been shown to play an important role in regulating NK cell activity and licensing in mice, by Ly49 interaction in *cis* (195, 196). In agreement, evidences in literature indicate that KIR:HLA interactions could occur both in *trans* and in *cis* in humans too. While the HLA-I trans-presentation seems to be mainly involved in NK cell education, the *cis* interaction in humans could play a major role in the maintenance of NK cell effector potential (190). However, since, unlike Ly49, KIRs do not have a flexible stalk, it has been proposed that this *cis* interaction between HLA-C and KIR2DL could occur in endosomes rather than on the cell surface (197).

Each individual shows a highly stochastic but tolerant HLA-I specific repertoire of iNKRs, which can be shaped by the subject-specific immunological history. These phenomena are regulated by several “licensing” iKIRs, that recognize HLA-A/B/C, and by CD94/NKG2A, that binds HLA-E (9, 198, 199). During NK cell maturation, the NKR repertoire is selected to the expression of at least one iNKRs specific for self-HLA-I haplotype on each mature NK cell. This makes NK cells able to recognize target cells, thus avoiding autoreactivity (200). CD94/NKG2A is the first HLA-I-specific iNKR expressed on differentiating NK cells prior to the appearance of KIRs. Indeed, it is present on CD56^br^ NK cells and to a less extent on CD56^dim^ NK cells (201). Moreover, *in vitro* differentiating NK cells from immature post-natal thymocytes express high levels of CD94/NKG2A that prevents the lysis of autologous cells expressing self-HLA-I alleles (202). However, it is widely accepted that iKIR^pos^ cells represent the main subset of “educated” NK lymphocytes. Indeed, during NK cell differentiation, the surface levels of CD94/NKG2A decrease while the expression of KIRs increases only on terminally differentiated CD56^dim^ NK cells (201). In this context, the human KIR gene family shows a certain degree of diversity due to both the high variability of KIR gene contents and allelic polymorphisms (203, 204). KIR and HLA genes are located on different chromosomes, and are inherited independently. This phenomenon might affect the selective evolutionary pressure as well as the NK cell-mediated susceptibility toward infections and diseases, as it is possible that KIR genes can be inherited in the absence of the cognate HLA ligand. Moreover, as previously mentioned, only NK cells expressing at least one KIR can be considered fully “licensed” (200, 205).

Although the mechanism tuning the process of NK cell education has not yet been fully disclosed, NK cell responsiveness is acquired in a finely regulated manner through KIR–KIR ligand interactions during development and several working hypotheses are currently being discussed on this matter. The first one relies on the concept of “arming” in which a given iKIR recognizes its cognate self-HLA-I allele, thus allowing the fully maturation of NK cells. In this regard, an NK cell expressing more than one iKIR should receive a stronger inhibitory signal, but this cell should also mediate a more potent alloreactivity when encountering a non-self-target (206). An opposite theory is instead based on the idea that NK cells expressing iKIRs mismatched with self HLA-I alleles are not clonally deleted, but are rather kept “unlicensed” both in PB and tissues in a state of hypo-responsiveness to ensure self-tolerance (194, 207, 208). This so called “disarming” working hypothesis states that, in the absence of self-iKIRs, the chronic stimulation of a still undetermined aNKR is associated with NK cell anergy (207).

An additional iNKR involved in NK cell education is ILT2/LIR1, which recognizes HLA-G and other shared epitopes present in all human HLA-I molecules. It has been shown that the expression of LIR1 by NK cells is able to prevent the secretion of IFN-γ (199). This iNKR appears to be expressed by mature NK cells and its surface level increases upon cytokine stimulation or HCMV infection, thus representing a possible escape mechanism from NK cell immune-surveillance (209–211). Moreover, the LIR1-mediated inhibition of NK cell effector-functions has been proposed to be also important in regulating the maternal-fetal immune tolerance during pregnancy (212).

Besides HLA-I specific iNKRs, several additional surface molecules have been reported to be involved in NK cell licensing to prevent their cell activation against self-cells. These additional mechanisms likely ensure a multi-layered and complementary system of immune tolerance and education of NK cells. In this context, the appearance of NKp46 and NKp30 before HLA-I specific iNKRs during development could ensure an HLA-I independent self-tolerance at early stages of NK cell differentiation (198, 213, 214). This hypothesis is also supported by clinical evidence in human HLA-I-deficient individuals, in which NK cells do not kill autologous cells although the engagement of HLA-specific iNKRs is either impaired or lacking (215, 216). In line with this last theory, it has been demonstrated that 2B4 could be involved in NK cell education by being expressed early on the CD34^pos^ NKPs. As a matter of fact, although 2B4 is an aNKR in mature NK cells, it can exert an inhibitory function when expressed on immature NK cells (198, 213).

Finally, another mechanism possibly preventing NK cell autoreactivity relies on the differential/asynchronous expression of NK cell receptors and ligands. A classic example is the expression of NKG2D, an aNKR able of modulating NK cell receptor activation with different thresholds (217). In adults, it has an important role in eliminate potentially dangerous cells expressing NKG2D ligands including tumor-transformed and viral-infected target cells. On the contrary, NKG2D is not expressed in embryonic life, when the soluble and exosome-bound ligands MIC-A and MIC-B are produced by human placenta. This mechanism thus contributes to prevent the activation of mother NK cells against the fetus (218, 219).

## Concluding Remarks

Although our current knowledge on the mechanisms tuning human NK cell ontogenesis greatly advanced over the past 2 decades, several questions still remain unanswered. In particular, the signals and pathways involved in NK cell development in SLTs and in other anatomic compartments remain to be clarified. Furthermore, the intracellular processes by which an NK cell is able to discern between self and non-self are still elusive. Emerging evidence from high-throughput technologies highlighted that NK cell diversity is more complex than expected and it is determined by genetic and environmental determinants. Thus, it is possible that this phenotypic NK cell diversity and apparent redundancy, within the same tissue and between the different tissues, could be the result of NK cell plasticity and could mirror the different NK cell functional properties rather than mere developmental intermediates.

Future efforts in understanding NK cell differentiation, effector-functions and heterogeneity in both physiological and pathological conditions will provide insights for the prevention and the treatment of human diseases. In particular, a better understanding of NK cell development in malignancies and other diseases will facilitate the design and implementation of NK cell-mediated immunotherapies.

## Author Contributions

CD, JM, and DM wrote the manuscript and approved the final version.

### Conflict of Interest Statement

The authors declare that the research was conducted in the absence of any commercial or financial relationships that could be construed as a potential conflict of interest.

## References

[1] Rosenberg EB Herberman RB Levine PH Halterman RH McCoy JL Wunderlich JR. Lymphocyte cytotoxicity reactions to leukemia-associated antigens in identical twins. Int J Cancer. (1972) 9:648–58. 10.1002/ijc.2910090323 4513057

[2] Agaugue S Marcenaro E Ferranti B Moretta L Moretta A. Human natural killer cells exposed to IL-2, IL-12, IL-18, or IL-4 differently modulate priming of naive T cells by monocyte-derived dendritic cells. Blood. (2008) 112:1776–83. 10.1182/blood-2008-02-135871 18579793

[3] Vitale M Della Chiesa M Carlomagno S Pende D Arico M Moretta L. NK-dependent DC maturation is mediated by TNFα and IFNγ released upon engagement of the NKp30 triggering receptor. Blood. (2005) 106:566–71. 10.1182/blood-2004-10-4035 15784725

[4] Moretta A. The dialogue between human natural killer cells and dendritic cells. Curr Opin Immunol. (2005) 17:306–11. 10.1016/j.coi.2005.03.004 15886122

[5] Mattiola I Pesant M Tentorio PF Molgora M Marcenaro E Lugli E. Priming of human resting NK cells by autologous M1 macrophages via the engagement of IL-1β, IFN-β, and IL-15 pathways. J Immunol. (2015) 195:2818–28. 10.4049/jimmunol.1500325 26276870

[6] Moretta A Bottino C Vitale M Pende D Cantoni C Mingari MC. Activating receptors and coreceptors involved in human natural killer cell-mediated cytolysis. Ann Rev Immunol. (2001) 19:197–223. 10.1146/annurev.immunol.19.1.197 11244035

[7] Lanier LL. Up on the tightrope: natural killer cell activation and inhibition. Nat Immunol. (2008) 9:495–502. 10.1038/ni1581 18425106 PMC2669298

[8] Vivier E Tomasello E Baratin M Walzer T Ugolini S. Functions of natural killer cells. Nat Immunol. (2008) 9:503–10. 10.1038/ni1582 18425107

[9] Moretta A Bottino C Vitale M Pende D Biassoni R Mingari MC. Receptors for HLA class-I molecules in human natural killer cells. Annu Rev Immunol. (1996) 14:619–48. 10.1146/annurev.immunol.14.1.619 8717527

[10] Sivori S Vitale M Bottino C Marcenaro E Sanseverino L Parolini S. CD94 functions as a natural killer cell inhibitory receptor for different HLA class I alleles: identification of the inhibitory form of CD94 by the use of novel monoclonal antibodies. Eur J Immunol. (1996) 26:2487–92. 10.1002/eji.1830261032 8898964

[11] Moretta L Moretta A. Unravelling natural killer cell function: triggering and inhibitory human NK receptors. EMBO J. (2004) 23:255–9. 10.1038/sj.emboj.7600019 14685277 PMC1271745

[12] Karre K. Natural killer cell recognition of missing self. Nat Immunol. (2008) 9:477–80. 10.1038/ni0508-477 18425103

[13] Moretta L Ciccone E Moretta A Hoglund P Ohlen C Karre K Allorecognition by NK cells: nonself or no self? Immunol Today. (1992) 13:300–6. 10.1016/0167-5699(92)90042-6 1380815

[14] Del Zotto G Marcenaro E Vacca P Sivori S Pende D Della Chiesa M. Markers and function of human NK cells in normal and pathological conditions. Cytometry B Clin Cytom. (2017) 92:100–14. 10.1002/cyto.b.21508 28054442

[15] Cooper MA Fehniger TA Caligiuri MA. The biology of human natural killer-cell subsets. Trends Immunol. (2001) 22:633–40. 10.1016/S1471-4906(01)02060-9 11698225

[16] Della Chiesa M Sivori S Carlomagno S Moretta L Moretta A. Activating KIRs and NKG2C in viral infections: toward NK cell memory? Front Immunol. (2015) 6:573. 10.3389/fimmu.2015.00573 26617607 PMC4638145

[17] Gondois-Rey F Granjeaud S Rouillier P Rioualen C Bidaut G Olive D. Multi-parametric cytometry from a complex cellular sample: improvements and limits of manual versus computational-based interactive analyses. Cytometry A. (2016) 89:480–90. 10.1002/cyto.a.22850 27059253

[18] Horowitz A Strauss-Albee DM Leipold M Kubo J Nemat-Gorgani N Dogan OC. Genetic and environmental determinants of human NK cell diversity revealed by mass cytometry. Sci Transl Med. (2013) 5:208ra145. 10.1126/scitranslmed.3006702 24154599 PMC3918221

[19] Lugli E Hudspeth K Roberto A Mavilio D. Tissue-resident and memory properties of human T-cell and NK-cell subsets. Eur J Immunol. (2016) 46:1809–17. 10.1002/eji.201545702 27431095

[20] Mikulak J Bruni E Oriolo F Di Vito C Mavilio D. Hepatic natural killer cells: organ-specific sentinels of liver immune homeostasis and physiopathology. Front Immunol. (2019) 10:946. 10.3389/fimmu.2019.00946 31114585 PMC6502999

[21] Bjorkstrom NK Ljunggren HG Michaelsson J. Emerging insights into natural killer cells in human peripheral tissues. Nat Rev Immunol. (2016) 16:310–20. 10.1038/nri.2016.34 27121652

[22] Yu J Freud AG Caligiuri MA. Location and cellular stages of natural killer cell development. Trends Immunol. (2013) 34:573–82. 10.1016/j.it.2013.07.005 24055329 PMC3852183

[23] Colucci F Caligiuri MA Di Santo JP. What does it take to make a natural killer? Nat Rev Immunol. (2003) 3:413–25. 10.1038/nri1088 12766763

[24] Godin I Cumano A. The hare and the tortoise: an embryonic haematopoietic race. Nat Rev Immunol. (2002) 2:593–604. 10.1038/nri857 12154378

[25] Freud AG Caligiuri MA. Human natural killer cell development. Immunol Rev. (2006) 214:56–72. 10.1111/j.1600-065X.2006.00451.x 17100876

[26] Freud AG Becknell B Roychowdhury S Mao HC Ferketich AK Nuovo GJ. A human CD34(+) subset resides in lymph nodes and differentiates into CD56bright natural killer cells. Immunity. (2005) 22:295–304. 10.1016/j.immuni.2005.01.013 15780987

[27] Male V Hughes T McClory S Colucci F Caligiuri MA Moffett A. Immature NK cells, capable of producing IL-22, are present in human uterine mucosa. J Immunol. (2010) 185:3913–8. 10.4049/jimmunol.1001637 20802153 PMC3795409

[28] Vacca P Vitale C Montaldo E Conte R Cantoni C Fulcheri E. CD34+ hematopoietic precursors are present in human decidua and differentiate into natural killer cells upon interaction with stromal cells. Proc Natl Acad Sci USA. (2011) 108:2402–7. 10.1073/pnas.1016257108 21248224 PMC3038730

[29] Moroso V Famili F Papazian N Cupedo T van der Laan LJ Kazemier G Metselaar HJ. NK cells can generate from precursors in the adult human liver. Eur J Immunol. (2011) 41:3340–50. 10.1002/eji.201141760 21830211

[30] Ferlazzo G Thomas D Lin SL Goodman K Morandi B Muller WA. The abundant NK cells in human secondary lymphoid tissues require activation to express killer cell Ig-like receptors and become cytolytic. J Immunol. (2004) 172:1455–62. 10.4049/jimmunol.172.3.1455 14734722

[31] Hao QL George AA Zhu J Barsky L Zielinska E Wang X. Human intrathymic lineage commitment is marked by differential CD7 expression: identification of CD7-lympho-myeloid thymic progenitors. Blood. (2008) 111:1318–26. 10.1182/blood-2007-08-106294 17959857 PMC2214748

[32] McLean-Tooke Barge D Spickett GP Gennery AR. Immunologic defects in 22q11.2 deletion syndrome. J Allergy Clin Immunol. (2008) 122:362–7. 10.1016/j.jaci.2008.03.033 18485468

[33] Ramos SB Garcia AB Viana SR Voltarelli JC Falcao RP. Phenotypic and functional evaluation of natural killer cells in thymectomized children. Clin Immunol Immunopathol. (1996) 81:277–81. 10.1006/clin.1996.0189 8938105

[34] Lalle M Minellli M Tarantini P Marino M Cerasoli V Facciolo F. Cellular and humoral immune alterations in thymectomized patients for thymoma. Ann Hematol. (2009) 88:847–53. 10.1007/s00277-008-0693-3 19165484

[35] Passlick B Izbicki JR Waydhas C Nast-Kolb D Schweiberer L Ziegler-Heitbrock HW Posttraumatic splenectomy does not influence human peripheral blood mononuclear cell subsets. J Clin Lab Immunol. (1991) 34:157–61. 1668282

[36] Golden-Mason L O'Farrelly C. Having it all? Stem cells, haematopoiesis and lymphopoiesis in adult human liver. Immunol Cell Biol. (2002) 80:45–51. 10.1046/j.1440-1711.2002.01066.x 11869362

[37] Moroso V Metselaar HJ Mancham S Tilanus HW Eissens D van der Meer A. Liver grafts contain a unique subset of natural killer cells that are transferred into the recipient after liver transplantation. Liver Transpl. (2010) 16:895–908. 10.1002/lt.22080 20583081

[38] Hudspeth K Donadon M Cimino M Pontarini E Tentorio P Preti M. Human liver-resident CD56(bright)/CD16(neg) NK cells are retained within hepatic sinusoids via the engagement of CCR5 and CXCR6 pathways. J Autoimmun. (2016) 66:40–50. 10.1016/j.jaut.2015.08.011 26330348 PMC4718768

[39] Marquardt N Beziat V Nystrom S Hengst J Ivarsson MA Kekalainen E. Cutting edge: identification and characterization of human intrahepatic CD49a+ NK cells. J Immunol. (2015) 194:2467–71. 10.4049/jimmunol.1402756 25672754

[40] Santoni A Carlino C Gismondi A. Uterine NK cell development, migration and function. Reprod Biomed Online. (2008) 16:202–10. 10.1016/S1472-6483(10)60575-5 18284874

[41] Vacca P Cantoni C Prato C Fulcheri E Moretta A Moretta L. Regulatory role of NKp44, NKp46, DNAM-1 and NKG2D receptors in the interaction between NK cells and trophoblast cells. Evidence for divergent functional profiles of decidual versus peripheral NK cells. Int Immunol. (2008) 20:1395–405. 10.1093/intimm/dxn105 18815119

[42] Moffett A Colucci F. Uterine NK cells: active regulators at the maternal-fetal interface. J Clin Invest. (2014) 124:1872–9. 10.1172/JCI68107 24789879 PMC4001528

[43] Vacca P Moretta L Moretta A Mingari MC. Origin, phenotype and function of human natural killer cells in pregnancy. Trends Immunol. (2011) 32:517–23. 10.1016/j.it.2011.06.013 21889405

[44] Glover LE Crosby D Thiruchelvam U Harmon C Chorcora CN Wingfield MB. Uterine natural killer cell progenitor populations predict successful implantation in women with endometriosis-associated infertility. Am J Reprod Immunol. (2018) 79. 10.1111/aji.12817 29380456

[45] Scoville SD Freud AG Caligiuri MA. Cellular pathways in the development of human and murine innate lymphoid cells. Curr Opin Immunol. (2018) 56:100–6. 10.1016/j.coi.2018.11.003 30579240 PMC7285385

[46] Di Santo JP. Natural killer cell developmental pathways: a question of balance. Annu Rev Immunol. (2006) 24:257–86. 10.1146/annurev.immunol.24.021605.090700 16551250

[47] Freud AG Yokohama A Becknell B Lee MT Mao HC Ferketich AK. Evidence for discrete stages of human natural killer cell differentiation *in vivo*. J Exp Med. (2006) 203:1033–43. 10.1084/jem.20052507 16606675 PMC2118285

[48] Abel AM Yang C Thakar MS Malarkannan S. Natural killer cells: development, maturation, and clinical utilization. Front Immunol. (2018) 9:1869. 10.3389/fimmu.2018.01869 30150991 PMC6099181

[49] Rosmaraki EE Douagi I Roth C Colucci F Cumano A Di Santo JP. Identification of committed NK cell progenitors in adult murine bone marrow. Eur J Immunol. (2001) 31:1900–9. 10.1002/1521-4141(200106)31:6<1900::AID-IMMU1900>3.0.CO;2-M 11433387

[50] Becknell B Caligiuri MA. Interleukin-2, interleukin-15, and their roles in human natural killer cells. Adv Immunol. (2005) 86:209–39. 10.1016/S0065-2776(04)86006-1 15705423

[51] Yu H Fehniger TA Fuchshuber P Thiel KS Vivier E Carson WE. Flt3 ligand promotes the generation of a distinct CD34(+) human natural killer cell progenitor that responds to interleukin-15. Blood. (1998) 92:3647–57. 9808558

[52] Scoville SD Mundy-Bosse BL Zhang MH Chen L Zhang X Keller KA. A progenitor cell expressing transcription factor RORgammat generates all human innate lymphoid cell subsets. Immunity. (2016) 44:1140–50. 10.1016/j.immuni.2016.04.007 27178467 PMC4893782

[53] Renoux VM Zriwil A Peitzsch C Michaelsson J Friberg D Soneji S. Identification of a human natural killer cell lineage-restricted progenitor in fetal and adult tissues. Immunity. (2015) 43:394–407. 10.1016/j.immuni.2015.07.011 26287684

[54] Chen L Youssef Y Robinson C Ernst GF Carson MY Young KA. CD56 expression marks human group 2 innate lymphoid cell divergence from a shared NK cell and group 3 innate lymphoid cell developmental pathway. Immunity. (2018) 49:464–76 e4. 10.1016/j.immuni.2018.08.010 30193847 PMC6148384

[55] Montaldo E Del Zotto G Della Chiesa M Mingari MC Moretta A De Maria A. Human NK cell receptors/markers: a tool to analyze NK cell development, subsets and function. Cytometry A. (2013) 83:702–13. 10.1002/cyto.a.22302 23650273

[56] Grzywacz B Kataria N Sikora M Oostendorp RA Dzierzak EA Blazar BR. Coordinated acquisition of inhibitory and activating receptors and functional properties by developing human natural killer cells. Blood. (2006) 108:3824–33. 10.1182/blood-2006-04-020198 16902150 PMC1895469

[57] Perussia B Chen Y Loza MJ. Peripheral NK cell phenotypes: multiple changing of faces of an adapting, developing cell. Mol Immunol. (2005) 42:385–95. 10.1016/j.molimm.2004.07.017 15607789

[58] Freud AG Keller KA Scoville SD Mundy-Bosse BL Cheng S Youssef Y. NKp80 defines a critical step during human natural killer cell development. Cell Rep. (2016) 16:379–91. 10.1016/j.celrep.2016.05.095 27373165 PMC4970225

[59] Vitale M Falco M Castriconi R Parolini S Zambello R Semenzato G. Identification of NKp80, a novel triggering molecule expressed by human NK cells. Eur J Immunol. (2001) 31:233–42. 10.1002/1521-4141(200101)31:1<233::AID-IMMU233>3.0.CO;2-4 11265639

[60] Romagnani C Juelke K Falco M Morandi B D'Agostino A Costa R. CD56brightCD16- killer Ig-like receptor- NK cells display longer telomeres and acquire features of CD56dim NK cells upon activation. J Immunol. (2007) 178:4947–55. 10.4049/jimmunol.178.8.4947 17404276

[61] Pesce S Squillario M Greppi M Loiacono F Moretta L Moretta A. New miRNA signature heralds human NK cell subsets at different maturation steps: involvement of miR-146a-5p in the regulation of KIR expression. Front Immunol. (2018) 9:2360. 10.3389/fimmu.2018.02360 30374356 PMC6196268

[62] Vitale M Della Chiesa M Carlomagno S Romagnani C Thiel A Moretta L. The small subset of CD56brightCD16- natural killer cells is selectively responsible for both cell proliferation and interferon-γ production upon interaction with dendritic cells. Eur J Immunol. (2004) 34:1715–22. 10.1002/eji.200425100 15162442

[63] Collins PL Cella M Porter SI Li S Gurewitz GL Hong HS. Gene regulatory programs conferring phenotypic identities to human NK cells. Cell. (2019) 176:348–60 e12. 10.1016/j.cell.2018.11.045 30595449 PMC6329660

[64] Bjorkstrom NK Riese P Heuts F Andersson S Fauriat C Ivarsson MA. Expression patterns of NKG2A, KIR, and CD57 define a process of CD56dim NK-cell differentiation uncoupled from NK-cell education. Blood. (2010) 116:3853–64. 10.1182/blood-2010-04-281675 20696944

[65] Lopez-Verges S Milush JM Pandey S York VA Arakawa-Hoyt J Pircher H. CD57 defines a functionally distinct population of mature NK cells in the human CD56dimCD16+ NK-cell subset. Blood. (2010) 116:3865–74. 10.1182/blood-2010-04-282301 20733159 PMC2981540

[66] Muccio L Bertaina A Falco M Pende D Meazza R Lopez-Botet M. Analysis of memory-like natural killer cells in human cytomegalovirus-infected children undergoing α*β*+T and B cell-depleted hematopoietic stem cell transplantation for hematological malignancies. Haematologica. (2016) 101:371–81. 10.3324/haematol.2015.134155 26659918 PMC4815729

[67] J.Pahl HW Cerwenka A Ni J Memory-like NK cells: remembering a previous activation by cytokines and NK Cell receptors. Front Immunol. (2018) 9:2796 10.3389/fimmu.2018.02796 30546366 PMC6279934

[68] Guma M Angulo A Vilches C Gomez-Lozano N Malats N Lopez-Botet M. Imprint of human cytomegalovirus infection on the NK cell receptor repertoire. Blood. (2004) 104:3664–71. 10.1182/blood-2004-05-2058 15304389

[69] Beziat V Liu LL Malmberg JA Ivarsson MA Sohlberg E Bjorklund AT. NK cell responses to cytomegalovirus infection lead to stable imprints in the human KIR repertoire and involve activating KIRs. Blood. (2013) 121:2678–88. 10.1182/blood-2012-10-459545 23325834 PMC3617633

[70] Hendricks DW Balfour HH Jr Dunmire SK Schmeling DO Hogquist KA Lanier LL Cutting edge: NKG2C(hi)CD57+ NK cells respond specifically to acute infection with cytomegalovirus and not Epstein-Barr virus. J Immunol. (2014) 192:4492–6. 10.4049/jimmunol.1303211 24740502 PMC4013527

[71] Lee J Zhang T Hwang I Kim A Nitschke L Kim M. Epigenetic modification and antibody-dependent expansion of memory-like NK cells in human cytomegalovirus-infected individuals. Immunity. (2015) 42:431–42. 10.1016/j.immuni.2015.02.013 25786175 PMC4537797

[72] Schlums H Cichocki F Tesi B Theorell J Beziat V Holmes TD. Cytomegalovirus infection drives adaptive epigenetic diversification of NK cells with altered signaling and effector function. Immunity. (2015) 42:443–56. 10.1016/j.immuni.2015.02.008 25786176 PMC4612277

[73] O'Sullivan TE Sun JC Lanier LL Natural killer cell memory. Immunity. (2015) 43:634–45. 10.1016/j.immuni.2015.09.013 26488815 PMC4621966

[74] Lugli E Marcenaro E Mavilio D. NK cell subset redistribution during the course of viral infections. Front Immunol. (2014) 5:390. 10.3389/fimmu.2014.00390 25177322 PMC4132273

[75] Luetke-Eversloh M Hammer Q Durek P Nordstrom K Gasparoni G Pink M. Human cytomegalovirus drives epigenetic imprinting of the IFNG locus in NKG2Chi natural killer cells. PLoS Pathog. (2014) 10:e1004441. 10.1371/journal.ppat.1004441 25329659 PMC4199780

[76] Paust S Gill HS Wang BZ Flynn MP Moseman EA Senman B. Critical role for the chemokine receptor CXCR6 in NK cell-mediated antigen-specific memory of haptens and viruses. Nat Immunol. (2010) 11:1127–35. 10.1038/ni.1953 20972432 PMC2982944

[77] Peng H Jiang X Chen Y Sojka DK Wei H Gao X. Liver-resident NK cells confer adaptive immunity in skin-contact inflammation. J Clin Invest. (2013) 123:1444–56. 10.1172/JCI66381 23524967 PMC3613925

[78] Mavilio D Lombardo G Benjamin J Kim D Follman D Marcenaro E. Characterization of CD56-/CD16+ natural killer (NK) cells: a highly dysfunctional NK subset expanded in HIV-infected viremic individuals. Proc Natl Acad Sci USA. (2005) 102:2886–91. 10.1073/pnas.0409872102 15699323 PMC549494

[79] Mikulak J Oriolo F Zaghi E Di Vito C Mavilio D. Natural killer cells in HIV-1 infection and therapy. AIDS. (2017) 31:2317–30. 10.1097/QAD.0000000000001645 28926399 PMC5892189

[80] Della Chiesa M Marcenaro E Sivori S Carlomagno S Pesce S Moretta A. Human NK cell response to pathogens. Semin Immunol. (2014) 26:152–60. 10.1016/j.smim.2014.02.001 24582551

[81] Muller-Durovic B Grahlert J Devine OP Akbar AN Hess C. CD56-negative NK cells with impaired effector function expand in CMV and EBV co-infected healthy donors with age. Aging. (2019) 11:724–40. 10.18632/aging.101774 30686790 PMC6366961

[82] Bjorkstrom NK Ljunggren HG Sandberg JK. CD56 negative NK cells: origin, function, and role in chronic viral disease. Trends Immunol. (2010) 31:401–6. 10.1016/j.it.2010.08.003 20829113

[83] Milush JM Lopez-Verges S York VA Deeks SG Martin JN Hecht FM. CD56negCD16(+) NK cells are activated mature NK cells with impaired effector function during HIV-1 infection. Retrovirology. (2013) 10:158. 10.1186/1742-4690-10-158 24351015 PMC3892122

[84] Zaghi E Calvi M Marcenaro E Mavilio D Di Vito C. Targeting NKG2A to elucidate natural killer cell ontogenesis and to develop novel immune-therapeutic strategies in cancer therapy. J Leukoc Biol. (2019) 105:1243–125. 10.1002/JLB.MR0718-300R 30645023

[85] Penack O Gentilini C Fischer L Asemissen AM Scheibenbogen C Thiel E. CD56dimCD16neg cells are responsible for natural cytotoxicity against tumor targets. Leukemia. (2005) 19:835–40. 10.1038/sj.leu.2403704 15744340

[86] Takahashi E Kuranaga N Satoh K Habu Y Shinomiya N Asano T Induction of CD16+ CD56bright NK cells with antitumour cytotoxicity not only from CD16- CD56bright NK cells but also from CD16- CD56dim NK cells. Scand J Immunol. (2007) 65:126–38. 10.1111/j.1365-3083.2006.01883.x 17257217

[87] Stabile H Nisti P Morrone S Pagliara D Bertaina A Locatelli F. Multifunctional human CD56 low CD16 low natural killer cells are the prominent subset in bone marrow of both healthy pediatric donors and leukemic patients. Haematologica. (2015) 100:489–98. 10.3324/haematol.2014.116053 25596273 PMC4380722

[88] Roberto A Di Vito C Zaghi E Mazza EMC Capucetti A Calvi M The early expansion of anergic NKG2A(pos)/CD56(dim)/CD16(neg) natural killer represents a therapeutic target in haploidentical hematopoietic stem cell transplantation. Haematologica. (2018) 103:1390–402. 10.3324/haematol.2017.186619 29700172 PMC6068034

[89] Vulpis E Stabile H Soriani A Fionda C Petrucci MT Mariggio E. Key role of the CD56^*low*^CD16^*low*^ natural killer cell subset in the recognition and killing of multiple myeloma cells. Cancers. (2018) 10:473. 10.3390/cancers10120473 30501078 PMC6317053

[90] Helena S Paolo N Giovanna P Cinzia F Daria P Letizia PB Reconstitution of multifunctional CD56(low)CD16(low) natural killer cell subset in children with acute leukemia given α/β T cell-depleted HLA-haploidentical haematopoietic stem cell transplantation. Oncoimmunology. (2017) 6:e1342024 10.1080/2162402X.2017.1342024 28932646 PMC5599095

[91] Wu Y Tian Z Wei H. Developmental and functional control of natural killer cells by cytokines. Front Immunol. (2017) 8:930. 10.3389/fimmu.2017.00930 28824650 PMC5543290

[92] Lyman SD Jacobsen SE. c-kit ligand and Flt3 ligand: stem/progenitor cell factors with overlapping yet distinct activities. Blood. (1998) 91:1101–34. 9454740

[93] Mackarehtschian K Hardin JD Moore KA Boast S Goff SP Lemischka IR. Targeted disruption of the flk2/flt3 gene leads to deficiencies in primitive hematopoietic progenitors. Immunity. (1995) 3:147–61. 10.1016/1074-7613(95)90167-1 7621074

[94] Waskow C Paul S Haller C Gassmann M Rodewald HR. Viable c-Kit(W/W) mutants reveal pivotal role for c-kit in the maintenance of lymphopoiesis. Immunity. (2002) 17:277–88. 10.1016/S1074-7613(02)00386-2 12354381

[95] Colucci F Di Santo JP. The receptor tyrosine kinase c-kit provides a critical signal for survival, expansion, and maturation of mouse natural killer cells. Blood. (2000) 95:984–91. 10648413

[96] Sanchez-Correa B Bergua JM Pera A Campos C Arcos MJ Banas H. *In vitro* culture with Interleukin-15 leads to expression of activating receptors and recovery of natural killer cell function in acute myeloid leukemia patients. Front Immunol. (2017) 8:931. 10.3389/fimmu.2017.00931 28824651 PMC5545593

[97] Kennedy MK Glaccum M Brown SN Butz EA Viney JL Embers M. Reversible defects in natural killer and memory CD8 T cell lineages in interleukin 15-deficient mice. J Exp Med. (2000) 191:771–80. 10.1084/jem.191.5.771 10704459 PMC2195858

[98] Lodolce JP Boone DL Chai S Swain RE Dassopoulos T Trettin S. IL-15 receptor maintains lymphoid homeostasis by supporting lymphocyte homing and proliferation. Immunity. (1998) 9:669–76. 10.1016/S1074-7613(00)80664-0 9846488

[99] Gilmour KC Fujii H Cranston T Davies EG Kinnon C Gaspar HB. Defective expression of the interleukin-2/interleukin-15 receptor β subunit leads to a natural killer cell-deficient form of severe combined immunodeficiency. Blood. (2001) 98:877–9. 10.1182/blood.V98.3.877 11468191

[100] Vosshenrich CA Ranson T Samson SI Corcuff E Colucci F Rosmaraki EE. Roles for common cytokine receptor γ-chain-dependent cytokines in the generation, differentiation, and maturation of NK cell precursors and peripheral NK cells *in vivo*. J Immunol. (2005) 174:1213–21. 10.4049/jimmunol.174.3.1213 15661875

[101] Williams NS Klem J Puzanov IJ Sivakumar PV Schatzle JD Bennett M. Natural killer cell differentiation: insights from knockout and transgenic mouse models and *in vitro* systems. Immunol Rev. (1998) 165:47–61. 10.1111/j.1600-065X.1998.tb01229.x 9850851

[102] Boulanger MJ Garcia KC. Shared cytokine signaling receptors: structural insights from the gp130 system. Adv Protein Chem. (2004) 68:107–46. 10.1016/S0065-3233(04)68004-1 15500860

[103] Kundig TM Schorle H Bachmann MF Hengartner H Zinkernagel RM Horak I. Immune responses in interleukin-2-deficient mice. Science. (1993) 262:1059–61. 10.1126/science.8235625 8235625

[104] Kasaian MT Whitters MJ Carter LL Lowe LD Jussif JM Deng B. IL-21 limits NK cell responses and promotes antigen-specific T cell activation: a mediator of the transition from innate to adaptive immunity. Immunity. (2002) 16:559–69. 10.1016/S1074-7613(02)00295-9 11970879

[105] Maki K Sunaga S Komagata Y Kodaira Y Mabuchi A Karasuyama H. Interleukin 7 receptor-deficient mice lack gammadelta T cells. Proc Natl Acad Sci USA. (1996) 93:7172–7. 10.1073/pnas.93.14.7172 8692964 PMC38955

[106] von Freeden-Jeffry U Vieira P Lucian LA McNeil T Burdach SE Murray R Lymphopenia in interleukin (IL)-7 gene-deleted mice identifies IL-7 as a nonredundant cytokine. J Exp Med. (1995) 181:1519–26. 10.1084/jem.181.4.1519 7699333 PMC2191954

[107] Mrozek E Anderson P Caligiuri MA. Rolef of interleukin-15 in the development of human CD56+ natural killer cells from CD34+ hematopoietic progenitor cells. Blood. (1996) 87:2632–40. 8639878

[108] Burkett PR Koka R Chien M Chai S Boone DL Ma A. Coordinate expression and trans presentation of interleukin (IL)-15Rα and IL-15 supports natural killer cell and memory CD8+ T cell homeostasis. J Exp Med. (2004) 200:825–34. 10.1084/jem.20041389 15452177 PMC2213280

[109] Dubois S Mariner J Waldmann TA Tagaya Y. IL-15Rα recycles and presents IL-15 In trans to neighboring cells. Immunity. (2002) 17:537–47. 10.1016/S1074-7613(02)00429-6 12433361

[110] Huntington ND Legrand N Alves NL Jaron B Weijer K Plet A. IL-15 trans-presentation promotes human NK cell development and differentiation *in vivo*. J Exp Med. (2009) 206:25–34. 10.1084/jem.20082013 19103877 PMC2626663

[111] Ghoreschi K Laurence A O'Shea JJ. Janus kinases in immune cell signaling. Immunol Rev. (2009) 228:273–87. 10.1111/j.1600-065X.2008.00754.x 19290934 PMC2782696

[112] Smith GA Uchida K Weiss A Taunton J. Essential biphasic role for JAK3 catalytic activity in IL-2 receptor signaling. Nat Chem Biol. (2016) 12:373–9. 10.1038/nchembio.2056 27018889 PMC4837022

[113] Suzuki K Nakajima H Saito Y Saito T Leonard WJ Iwamoto I. Janus kinase 3 (Jak3) is essential for common cytokine receptor γ chain (γc)-dependent signaling: comparative analysis of γc, Jak3, and γc and Jak3 double-deficient mice. Int Immunol. (2000) 12:123–32. 10.1093/intimm/12.2.123 10653847

[114] Cooper MA Bush JE Fehniger TA VanDeusen JB Waite RE Liu Y. *In vivo* evidence for a dependence on interleukin 15 for survival of natural killer cells. Blood. (2002) 100:3633–8. 10.1182/blood-2001-12-0293 12393617

[115] Moriggl R Topham DJ Teglund S Sexl V McKay C Wang D. Stat5 is required for IL-2-induced cell cycle progression of peripheral T cells. Immunity. (1999) 10:249–59. 10.1016/S1074-7613(00)80025-4 10072077

[116] Eckelhart E Warsch W Zebedin E Simma O Stoiber D Kolbe T. A novel Ncr1-Cre mouse reveals the essential role of STAT5 for NK-cell survival and development. Blood. (2011) 117:1565–73. 10.1182/blood-2010-06-291633 21127177

[117] Bernasconi A Marino R Ribas A Rossi J Ciaccio M Oleastro M. Characterization of immunodeficiency in a patient with growth hormone insensitivity secondary to a novel STAT5b gene mutation. Pediatrics. (2006) 118:e1584–92. 10.1542/peds.2005-2882 17030597

[118] Copeland NG Gilbert DJ Schindler C Zhong Z Wen Z Darnell JE Jr. Distribution of the mammalian Stat gene family in mouse chromosomes. Genomics. (1995) 29:225–8. 10.1006/geno.1995.1235 8530075

[119] Yang M Li D Chang Z Yang Z Tian Z Dong Z. PDK1 orchestrates early NK cell development through induction of E4BP4 expression and maintenance of IL-15 responsiveness. J Exp Med. (2015) 212:253–65. 10.1084/jem.20141703 25624444 PMC4322053

[120] Yokota Y Mansouri A Mori S Sugawara S Adachi S Nishikawa S. Development of peripheral lymphoid organs and natural killer cells depends on the helix-loop-helix inhibitor Id2. Nature. (1999) 397:702–6. 10.1038/17812 10067894

[121] Boos MD Yokota Y Eberl G Kee BL. Mature natural killer cell and lymphoid tissue-inducing cell development requires Id2-mediated suppression of E protein activity. J Exp Med. (2007) 204:1119–30. 10.1084/jem.20061959 17452521 PMC2118569

[122] Parrish-Novak J Dillon SR Nelson A Hammond A Sprecher C Gross JA. Interleukin 21 and its receptor are involved in NK cell expansion and regulation of lymphocyte function. Nature. (2000) 408:57–63. 10.1038/35040504 11081504

[123] Sivori S Cantoni C Parolini S Marcenaro E Conte R Moretta L. IL-21 induces both rapid maturation of human CD34+ cell precursors towards NK cells and acquisition of surface killer Ig-like receptors. Eur J Immunol. (2003) 33:3439–47. 10.1002/eji.200324533 14635054

[124] Ni X Fu B Zhang J Sun R Tian Z Wei H. Cytokine-based generation of CD49a(+)Eomes(−/+) natural killer cell subsets. Front Immunol. (2018) 9:2126. 10.3389/fimmu.2018.02126 30319610 PMC6167425

[125] Sojka DK Plougastel-Douglas B Yang L Pak-Wittel MA Artyomov MN Ivanova Y. Tissue-resident natural killer (NK) cells are cell lineages distinct from thymic and conventional splenic NK cells. Elife. (2014) 3:e01659. 10.7554/eLife.01659 24714492 PMC3975579

[126] Hudspeth K Pontarini E Tentorio P Cimino M Donadon M Torzilli G. The role of natural killer cells in autoimmune liver disease: a comprehensive review. J Autoimmun. (2013) 46:55–65. 10.1016/j.jaut.2013.07.003 23880068

[127] Lehmann D Spanholtz J Sturtzel C Tordoir M Schlechta B Groenewegen D. IL-12 directs further maturation of *ex vivo* differentiated NK cells with improved therapeutic potential. PLoS ONE. (2014) 9:e87131. 10.1371/journal.pone.0087131 24498025 PMC3909052

[128] Ohs I van den Broek M. Nussbaum K Munz C Arnold SJ Quezada SA. Interleukin-12 bypasses common gamma-chain signalling in emergency natural killer cell lymphopoiesis. Nat Commun. (2016) 7:13708. 10.1038/ncomms13708 27982126 PMC5172358

[129] Geary CD Sun JC. Memory responses of natural killer cells. Semin Immunol. (2017) 31:11–19. 10.1016/j.smim.2017.08.012 28863960 PMC5724965

[130] Sun JC Madera S Bezman NA Beilke JN Kaplan MH Lanier LL. Proinflammatory cytokine signaling required for the generation of natural killer cell memory. J Exp Med. (2012) 209:947–54. 10.1084/jem.20111760 22493516 PMC3348098

[131] Hammer Q Ruckert T Borst EM Dunst J Haubner A Durek P. Peptide-specific recognition of human cytomegalovirus strains controls adaptive natural killer cells. Nat Immunol. (2018) 19:453–63. 10.1038/s41590-018-0082-6 29632329

[132] Rolle A Pollmann J Ewen EM Le VT Halenius A Hengel H. IL-12-producing monocytes and HLA-E control HCMV-driven NKG2C+ NK cell expansion. J Clin Invest. (2014) 124:5305–16. 10.1172/JCI77440 25384219 PMC4348979

[133] Cooper MA Elliott JM Keyel PA Yang L Carrero JA Yokoyama WM. Cytokine-induced memory-like natural killer cells. Proc Natl Acad Sci USA. (2009) 106:1915–9. 10.1073/pnas.0813192106 19181844 PMC2644138

[134] Romee R Schneider SE Leong JW Chase JM Keppel CR Sullivan RP. Cytokine activation induces human memory-like NK cells. Blood. (2012) 120:4751–60. 10.1182/blood-2012-04-419283 22983442 PMC3520618

[135] Romee R Rosario M Berrien-Elliott MM Wagner JA Jewell BA Schappe T. Cytokine-induced memory-like natural killer cells exhibit enhanced responses against myeloid leukemia. Sci Transl Med. (2016) 8:357ra123. 10.1126/scitranslmed.aaf2341 27655849 PMC5436500

[136] Boos MD Ramirez K Kee BL. Extrinsic and intrinsic regulation of early natural killer cell development. Immunol Res. (2008) 40:193–207. 10.1007/s12026-007-8006-9 18266115

[137] Male V Brady HJ. Transcriptional control of NK cell differentiation and function. Curr Top Microbiol Immunol. (2014) 381:173–87. 10.1007/82_2014_376 24850220

[138] Ramirez K Chandler KJ Spaulding C Zandi S Sigvardsson M Graves BJ. Gene deregulation and chronic activation in natural killer cells deficient in the transcription factor ETS1. Immunity. (2012) 36:921–32. 10.1016/j.immuni.2012.04.006 22608498 PMC3389314

[139] Nutt SL Metcalf D D'Amico A Polli M Wu L. Dynamic regulation of PU.1 expression in multipotent hematopoietic progenitors. J Exp Med. (2005) 201:221–31. 10.1084/jem.20041535 15657291 PMC2212785

[140] Carotta S Dakic A D'Amico A Pang SH Greig KT Nutt SL. The transcription factor PU.1 controls dendritic cell development and Flt3 cytokine receptor expression in a dose-dependent manner. Immunity. (2010) 32:628–41. 10.1016/j.immuni.2010.05.005 20510871

[141] Hollenhorst PC McIntosh LP Graves BJ. Genomic and biochemical insights into the specificity of ETS transcription factors. Annu Rev Biochem. (2011) 80:437–71. 10.1146/annurev.biochem.79.081507.103945 21548782 PMC5568663

[142] Barton K Muthusamy N Fischer C Ting CN Walunas TL. The Ets-1 transcription factor is required for the development of natural killer cells in mice. Immunity. (1998) 9:555–63. 10.1016/S1074-7613(00)80638-X 9806641

[143] Colucci F Samson SI DeKoter RP Lantz O Singh H Di Santo JP. Differential requirement for the transcription factor PU.1 in the generation of natural killer cells versus B and T cells. Blood. (2001) 97:2625–32. 10.1182/blood.V97.9.2625 11313251

[144] Lin JX Li P Liu D Jin HT He J Ata Ur Rasheed M, M. Critical role of STAT5 transcription factor tetramerization for cytokine responses and normal immune function. Immunity. (2012) 36:586–99. 10.1016/j.immuni.2012.02.017 22520852 PMC3551341

[145] Collins A Littman DR Taniuchi I. RUNX proteins in transcription factor networks that regulate T-cell lineage choice. Nat Rev Immunol. (2009) 9:106–15. 10.1038/nri2489 19165227 PMC4231139

[146] Ohno S Sato T Kohu K Takeda K Okumura K Satake M. Runx proteins are involved in regulation of CD122, Ly49 family and IFN-γ expression during NK cell differentiation. Int Immunol. (2008) 20:71–9. 10.1093/intimm/dxm120 18003603

[147] Aliahmad P Seksenyan A Kaye J. The many roles of TOX in the immune system. Curr Opin Immunol. (2012) 24:173–7. 10.1016/j.coi.2011.12.001 22209117 PMC3319641

[148] Tamura T Yanai H Savitsky D Taniguchi T. The IRF family transcription factors in immunity and oncogenesis. Annu Rev Immunol. (2008) 26:535–84. 10.1146/annurev.immunol.26.021607.090400 18303999

[149] Lohoff M Duncan GS Ferrick D Mittrucker HW Bischof S Prechtl S. Deficiency in the transcription factor interferon regulatory factor (IRF)-2 leads to severely compromised development of natural killer and T helper type 1 cells. J Exp Med. (2000) 192:325–36. 10.1084/jem.192.3.325 10934221 PMC2193225

[150] Gascoyne DM Long E Veiga-Fernandes H de Boer J Williams O Seddon B. The basic leucine zipper transcription factor E4BP4 is essential for natural killer cell development. Nat Immunol. (2009) 10:1118–24. 10.1038/ni.1787 19749763

[151] Kamizono S Duncan GS Seidel MG Morimoto A Hamada K Grosveld G. Nfil3/E4bp4 is required for the development and maturation of NK cells *in vivo*. J Exp Med. (2009) 206:2977–86. 10.1084/jem.20092176 19995955 PMC2806474

[152] Geiger TL Abt MC Gasteiger G Firth MA O'Connor MH Geary CD. Nfil3 is crucial for development of innate lymphoid cells and host protection against intestinal pathogens. J Exp Med. (2014) 211:1723–31. 10.1084/jem.20140212 25113970 PMC4144732

[153] Seillet C Rankin LC Groom JR Mielke LA Tellier J Chopin M. Nfil3 is required for the development of all innate lymphoid cell subsets. J Exp Med. (2014) 211:1733–40. 10.1084/jem.20140145 25092873 PMC4144736

[154] Male V Nisoli I Kostrzewski T Allan DS Carlyle JR Lord GM. The transcription factor E4bp4/Nfil3 controls commitment to the NK lineage and directly regulates Eomes and Id2 expression. J Exp Med. (2014) 211:635–42. 10.1084/jem.20132398 24663216 PMC3978281

[155] Seillet C Huntington ND Gangatirkar P Axelsson E Minnich M Brady HJ. Differential requirement for Nfil3 during NK cell development. J Immunol. (2014) 192:2667–76. 10.4049/jimmunol.1302605 24532575

[156] Crotta S Gkioka A Male V Duarte JH Davidson S Nisoli I The transcription factor E4BP4 is not required for extramedullary pathways of NK cell development. J Immunol. (2014) 192:2677–88. 10.4049/jimmunol.1302765 24534532 PMC3948112

[157] Delconte RB Shi W Sathe P Ushiki T Seillet C Minnich M. The Helix-loop-helix protein ID2 governs NK cell fate by tuning their sensitivity to Interleukin-15. Immunity. (2016) 44:103–15. 10.1016/j.immuni.2015.12.007 26795246

[158] Gordon SM Chaix J Rupp LJ Wu J Madera S Sun JC. The transcription factors T-bet and Eomes control key checkpoints of natural killer cell maturation. Immunity. (2012) 36:55–67. 10.1016/j.immuni.2011.11.016 22261438 PMC3381976

[159] Zhang J Marotel M Fauteux-Daniel S Mathieu AL Viel S Marcais A. T-bet and Eomes govern differentiation and function of mouse and human NK cells and ILC1. Eur J Immunol. (2018) 48:738–50. 10.1002/eji.201747299 29424438

[160] Jenne CN Enders A Rivera R Watson SR Bankovich AJ Pereira JP. T-bet-dependent S1P5 expression in NK cells promotes egress from lymph nodes and bone marrow. J Exp Med. (2009) 206:2469–81. 10.1084/jem.20090525 19808259 PMC2768857

[161] Pikovskaya O Chaix J Rothman NJ Collins A Chen YH Scipioni AM. Cutting edge: eomesodermin is sufficient to direct type 1 innate lymphocyte development into the conventional NK lineage. J Immunol. (2016) 196:1449–54. 10.4049/jimmunol.1502396 26792802 PMC4744497

[162] Townsend MJ Weinmann AS Matsuda JL Salomon R Farnham PJ Biron CA. T-bet regulates the terminal maturation and homeostasis of NK and Vα14i NKT cells. Immunity. (2004) 20:477–94. 10.1016/S1074-7613(04)00076-7 15084276

[163] Sciume G Hirahara K Takahashi H Laurence A Villarino AV Singleton KL. Distinct requirements for T-bet in gut innate lymphoid cells. J Exp Med. (2012) 209:2331–8. 10.1084/jem.20122097 23209316 PMC3526352

[164] Daussy C Faure F Mayol K Viel S Gasteiger G Charrier E. T-bet and Eomes instruct the development of two distinct natural killer cell lineages in the liver and in the bone marrow. J Exp Med. (2014) 211:563–77. 10.1084/jem.20131560 24516120 PMC3949572

[165] Gregoire C Chasson L Luci C Tomasello E Geissmann F Vivier E. The trafficking of natural killer cells. Immunol Rev. (2007) 220:169–82. 10.1111/j.1600-065X.2007.00563.x 17979846 PMC7165697

[166] Bozzano F Marras F Ascierto ML Cantoni C Cenderello G Dentone C. 'Emergency exit' of bone-marrow-resident CD34(+)DNAM-1(bright)CXCR4(+)-committed lymphoid precursors during chronic infection and inflammation. Nat Commun. (2015) 6:8109. 10.1038/ncomms9109 26436997 PMC4600731

[167] Inngjerdingen M Damaj B Maghazachi AA. Expression and regulation of chemokine receptors in human natural killer cells. Blood. (2001) 97:367–75. 10.1182/blood.V97.2.367 11154210

[168] Bellora F Castriconi R Dondero A Carrega P Mantovani A Ferlazzo G. Human NK cells and NK receptors. Immunol Lett. (2014) 161:168–73. 10.1016/j.imlet.2013.12.009 24361820

[169] Broxmeyer HE Orschell CM Clapp DW Hangoc G Cooper S Plett PA. Rapid mobilization of murine and human hematopoietic stem and progenitor cells with AMD3100, a CXCR4 antagonist. J Exp Med. (2005) 201:1307–18. 10.1084/jem.20041385 15837815 PMC2213145

[170] McDermott DH Liu Q Velez D Lopez L Anaya-O'Brien S Ulrick J. A phase 1 clinical trial of long-term, low-dose treatment of WHIM syndrome with the CXCR4 antagonist plerixafor. Blood. (2014) 123:2308–16. 10.1182/blood-2013-09-527226 24523241 PMC3983611

[171] Beider K Nagler A Wald O Franitza S Dagan-Berger M Wald H. Involvement of CXCR4 and IL-2 in the homing and retention of human NK and NK T cells to the bone marrow and spleen of NOD/SCID mice. Blood. (2003) 102:1951–8. 10.1182/blood-2002-10-3293 12730102

[172] Castriconi R Dondero A Bellora F Moretta L Castellano A Locatelli F. Neuroblastoma-derived TGF-β1 modulates the chemokine receptor repertoire of human resting NK cells. J Immunol. (2013) 190:5321–8. 10.4049/jimmunol.1202693 23576682

[173] Sciume G De Angelis G Benigni G Ponzetta A Morrone S Santoni A. CX3CR1 expression defines 2 KLRG1+ mouse NK-cell subsets with distinct functional properties and positioning in the bone marrow. Blood. (2011) 117:4467–75. 10.1182/blood-2010-07-297101 21364193

[174] Hamann I Unterwalder N Cardona AE Meisel C Zipp F Ransohoff RM. Analyses of phenotypic and functional characteristics of CX3CR1-expressing natural killer cells. Immunology. (2011) 133:62–73. 10.1111/j.1365-2567.2011.03409.x 21320123 PMC3088968

[175] Parolini S Santoro A Marcenaro E Luini W Massardi L Facchetti F. The role of chemerin in the colocalization of NK and dendritic cell subsets into inflamed tissues. Blood. (2007) 109:3625–32. 10.1182/blood-2006-08-038844 17202316

[176] Drouillard A Mathieu AL Marcais A Belot A Viel S Mingueneau M. S1PR5 is essential for human natural killer cell migration toward sphingosine-1 phosphate. J Allergy Clin Immunol. (2018) 141:2265–8 e1. 10.1016/j.jaci.2017.11.022 29248494

[177] Walzer T Chiossone L Chaix J Calver A Carozzo C Garrigue-Antar L. Natural killer cell trafficking *in vivo* requires a dedicated sphingosine 1-phosphate receptor. Nat Immunol. (2007) 8:1337–44. 10.1038/ni1523 17965716

[178] Della Chiesa M Sivori S Castriconi R Marcenaro E Moretta A. Pathogen-induced private conversations between natural killer and dendritic cells. Trends Microbiol. (2005) 13:128–36. 10.1016/j.tim.2005.01.006 15737731

[179] Fehniger TA Cooper MA Nuovo GJ Cella M Facchetti F Colonna M. CD56bright natural killer cells are present in human lymph nodes and are activated by T cell-derived IL-2: a potential new link between adaptive and innate immunity. Blood. (2003) 101:3052–7. 10.1182/blood-2002-09-2876 12480696

[180] Marcenaro E Cantoni C Pesce S Prato C Pende D Agaugue S. Uptake of CCR7 and acquisition of migratory properties by human KIR+ NK cells interacting with monocyte-derived DC or EBV cell lines: regulation by KIR/HLA-class I interaction. Blood. (2009) 114:4108–16. 10.1182/blood-2009-05-222265 19749090

[181] Mselle TF Meadows SK Eriksson M Smith JM Shen L Wira CR. Unique characteristics of NK cells throughout the human female reproductive tract. Clin Immunol. (2007) 124:69–76. 10.1016/j.clim.2007.04.008 17524808

[182] Ali TH Pisanti S Ciaglia E Mortarini R Anichini A Garofalo C. Enrichment of CD56(dim)KIR + CD57 + highly cytotoxic NK cells in tumour-infiltrated lymph nodes of melanoma patients. Nat Commun. (2014) 5:5639. 10.1038/ncomms6639 25472612 PMC4338526

[183] Lima M Leander M Santos M Santos AH Lau C Queiros ML. Chemokine receptor expression on normal blood CD56(+) NK-cells elucidates cell partners that comigrate during the innate and adaptive immune responses and identifies a transitional NK-cell population. J Immunol Res. (2015) 2015:839684. 10.1155/2015/839684 26543875 PMC4620293

[184] Pesce S Moretta L Moretta A Marcenaro E. Human NK cell subsets redistribution in pathological conditions: a role for CCR7 receptor. Front Immunol. (2016) 7:414. 10.3389/fimmu.2016.00414 27774094 PMC5053980

[185] Siewiera J Gouilly J Hocine HR Cartron G Levy C Al-Daccak R. Natural cytotoxicity receptor splice variants orchestrate the distinct functions of human natural killer cell subtypes. Nat Commun. (2015) 6:10183. 10.1038/ncomms10183 26666685 PMC4682172

[186] Cerdeira AS Rajakumar A Royle CM Lo A Husain Z Thadhani RI. Conversion of peripheral blood NK cells to a decidual NK-like phenotype by a cocktail of defined factors. J Immunol. (2013) 190:3939–48. 10.4049/jimmunol.1202582 23487420 PMC3742368

[187] Hadley GA Higgins JM. Integrin αEβ7: molecular features and functional significance in the immune system. Adv Exp Med Biol. (2014) 819:97–110. 10.1007/978-94-017-9153-3_7 25023170

[188] Peng H Tian Z. Diversity of tissue-resident NK cells. Semin Immunol. (2017) 31:3–10. 10.1016/j.smim.2017.07.006 28802693

[189] Moretta A. Natural killer cells and dendritic cells: rendezvous in abused tissues. Nat Rev Immunol. (2002) 2:957–64. 10.1038/nri956 12461568

[190] Boudreau JE Liu XR Zhao Z Zhang A Shultz LD Greiner DL Cell-extrinsic MHC class I molecule engagement augments human NK cell education programmed by cell-intrinsic MHC class immunity I. (2016) 45:280–91. 10.1016/j.immuni.2016.07.005 PMC5003427 27496730

[191] Boudreau JE Hsu KC. Natural killer cell education and the response to infection and cancer therapy: stay tuned. Trends Immunol. (2018) 39:222–39. 10.1016/j.it.2017.12.001 29397297 PMC6013060

[192] Yokoyama WM Kim S. Licensing of natural killer cells by self-major histocompatibility complex class. Immunol Rev I. (2006) 214:143–54. 10.1111/j.1600-065X.2006.00458.x 17100882

[193] Brodin P Hoglund P. Beyond licensing and disarming: a quantitative view on NK-cell education. Eur J Immunol. (2008) 38:2934–7. 10.1002/eji.200838760 18979511

[194] Kim S Poursine-Laurent J Truscott SM Lybarger L Song YJ Yang L. Licensing of natural killer cells by host major histocompatibility complex class I molecules. Nature. (2005) 436:709–13. 10.1038/nature03847 16079848

[195] Scarpellino L Oeschger F Guillaume P Coudert JD Levy F Leclercq G. Interactions of Ly49 family receptors with MHC class I ligands in trans and cis. J Immunol. (2007) 178:1277–84. 10.4049/jimmunol.178.3.1277 17237373

[196] Bessoles S Angelov GS Back J Leclercq G Vivier E Held W. Education of murine NK cells requires both cis and trans recognition of MHC class I molecules. J Immunol. (2013) 191:5044–51. 10.4049/jimmunol.1301971 24098052

[197] Li H Ivarsson MA Walker-Sperling VE Subleski J Johnson JK Wright PW. Identification of an elaborate NK-specific system regulating HLA-C expression. PLoS Genet. (2018) 14:e1007163. 10.1371/journal.pgen.1007163 29329284 PMC5785035

[198] Meazza R Falco M Marcenaro S Loiacono F Canevali P Bellora F. Inhibitory 2B4 contributes to NK cell education and immunological derangements in XLP1 patients. Eur J Immunol. (2017) 47:1051–61. 10.1002/eji.201646885 28386908

[199] Morel E Bellon T. HLA class I molecules regulate IFN-γ production induced in NK cells by target cells, viral products, or immature dendritic cells through the inhibitory receptor ILT2/CD85j. J Immunol. (2008) 181:2368–81. 10.4049/jimmunol.181.4.2368 18684926

[200] Orr MT Lanier LL. Natural killer cell education and tolerance. Cell. (2010) 142:847–56. 10.1016/j.cell.2010.08.031 20850008 PMC2945212

[201] Beziat V Descours B Parizot C Debre P Vieillard V. NK cell terminal differentiation: correlated stepwise decrease of NKG2A and acquisition of KIRs. PLoS ONE. (2010) 5:e11966. 10.1371/journal.pone.0011966 20700504 PMC2917352

[202] Mingari MC Vitale C Cantoni C Bellomo R Ponte M Schiavetti F. Interleukin-15-induced maturation of human natural killer cells from early thymic precursors: selective expression of CD94/NKG2-A as the only HLA class I-specific inhibitory receptor. Eur J Immunol. (1997) 27:1374–80. 10.1002/eji.1830270612 9209487

[203] Hsu KC Chida S Geraghty DE Dupont B. The killer cell immunoglobulin-like receptor (KIR) genomic region: gene-order, haplotypes and allelic polymorphism. Immunol Rev. (2002) 190:40–52. 10.1034/j.1600-065X.2002.19004.x 12493005

[204] Vilches C Parham P. KIR: diverse, rapidly evolving receptors of innate and adaptive immunity. Annu Rev Immunol. (2002) 20:217–51. 10.1146/annurev.immunol.20.092501.134942 11861603

[205] Falco M Moretta L Moretta A Bottino C. KIR and KIR ligand polymorphism: a new area for clinical applications? Tissue Antigens. (2013) 82:363–73. 10.1111/tan.12262 24498992

[206] Joncker NT Fernandez NC Treiner E Vivier E Raulet DH. NK cell responsiveness is tuned commensurate with the number of inhibitory receptors for self-MHC class I: the rheostat model. J Immunol. (2009) 182:4572–80. 10.4049/jimmunol.0803900 19342631 PMC2938179

[207] Elliott JM Yokoyama WM. Unifying concepts of MHC-dependent natural killer cell education. Trends Immunol. (2011) 32:364–72. 10.1016/j.it.2011.06.001 21752715 PMC3151350

[208] Thielens A Vivier E Romagne F. NK cell MHC class I specific receptors (KIR): from biology to clinical intervention. Curr Opin Immunol. (2012) 24:239–45. 10.1016/j.coi.2012.01.001 22264929

[209] Prod'homme V. Griffin C Aicheler RJ Wang EC McSharry BP Rickards CR. The human cytomegalovirus MHC class I homolog UL18 inhibits LIR-1+ but activates LIR-1- NK cells. J Immunol. (2007) 178:4473–81. 10.4049/jimmunol.178.7.4473 17372005 PMC2843079

[210] Li NL Fu L Uchtenhagen H Achour A Burshtyn DN. Cis association of leukocyte Ig-like receptor 1 with MHC class I modulates accessibility to antibodies and HCMV UL18. Eur J Immunol. (2013) 43:1042–52. 10.1002/eji.201242607 23348966

[211] Chapman TL Heikeman AP Bjorkman PJ. The inhibitory receptor LIR-1 uses a common binding interaction to recognize class I MHC molecules and the viral homolog UL18. Immunity. (1999) 11:603–13. 10.1016/S1074-7613(00)80135-1 10591185

[212] Ponte M Cantoni C Biassoni R Tradori-Cappai A Bentivoglio G Vitale C. Inhibitory receptors sensing HLA-G1 molecules in pregnancy: decidua-associated natural killer cells express LIR-1 and CD94/NKG2A and acquire p49, an HLA-G1-specific receptor. Proc Natl Acad Sci USA. (1999) 96:5674–9. 10.1073/pnas.96.10.5674 10318943 PMC21919

[213] Sivori S Falco M Marcenaro E Parolini S Biassoni R Bottino C. Early expression of triggering receptors and regulatory role of 2B4 in human natural killer cell precursors undergoing *in vitro* differentiation. Proc Natl Acad Sci USA. (2002) 99:4526–31. 10.1073/pnas.072065999 11917118 PMC123681

[214] Vacca P Pietra G Falco M Romeo E Bottino C Bellora F. Analysis of natural killer cells isolated from human decidua: evidence that 2B4 (CD244) functions as an inhibitory receptor and blocks NK-cell function. Blood. (2006) 108:4078–85. 10.1182/blood-2006-04-017343 16931625

[215] Vitale M Zimmer J Castriconi R Hanau D Donato L Bottino C. Analysis of natural killer cells in TAP2-deficient patients: expression of functional triggering receptors and evidence for the existence of inhibitory receptor(s) that prevent lysis of normal autologous cells. Blood. (2002) 99:1723–9. 10.1182/blood.V99.5.1723 11861289

[216] Zimmer J Donato L Hanau D Cazenave JP Tongio MM Moretta A. Activity and phenotype of natural killer cells in peptide transporter (TAP)-deficient patients (type I bare lymphocyte syndrome). J Exp Med. (1998) 187:117–22. 10.1084/jem.187.1.117 9419217 PMC2199183

[217] Wensveen FM Jelencic V Polic B. NKG2D: a master regulator of immune cell responsiveness. Front Immunol. (2018) 9:441. 10.3389/fimmu.2018.00441 29568297 PMC5852076

[218] Mincheva-Nilsson L Nagaeva O Chen T Stendahl U Antsiferova J Mogren I. Placenta-derived soluble MHC class I chain-related molecules down-regulate NKG2D receptor on peripheral blood mononuclear cells during human pregnancy: a possible novel immune escape mechanism for fetal survival. J Immunol. (2006) 176:3585–92. 10.4049/jimmunol.176.6.3585 16517727

[219] Hedlund M Stenqvist AC Nagaeva O Kjellberg L Wulff M Baranov V. Human placenta expresses and secretes NKG2D ligands via exosomes that down-modulate the cognate receptor expression: evidence for immunosuppressive function. J Immunol. (2009) 183:340–51. 10.4049/jimmunol.0803477 19542445

